# Unraveling an enhancer-silencer regulatory element showing epistatic interaction with a variant that escaped genome-wide association studies

**DOI:** 10.1016/j.xgen.2025.100889

**Published:** 2025-05-28

**Authors:** Mathieu Adjemout, Samia Nisar, Amélie Escandell, Romain Torres, Magali Torres, Hong Thu Nguyen Huu, Alassane Thiam, Iris Manosalva, Babacar Mbengue, Alioune Dieye, Véronique Adoue, Salvatore Spicuglia, Pascal Rihet, Sandrine Marquet

**Affiliations:** 1 Aix Marseille University, INSERM, TAGC, MarMaRa Institute, Marseille, France; 2 Infinity, Toulouse Institute for Infectious and Inflammatory Diseases, University of Toulouse, Inserm U1291, CNRS U5051 Toulouse, France; 3 Unité d'Immunogénétique, Institut Pasteur de Dakar, Dakar BP 220, Senegal; 4 Service d’Immunologie, Université Cheikh Anta Diop de Dakar, Dakar BP5005, Senegal; 5 CNRS, Marseille, France

**Keywords:** *cis*-regulatory elements, enhancer-silencer, dual function, epistatic interaction, functional variants, severe malaria, T cell activation, transcriptional regulation

## Abstract

Regulation of gene expression has recently been complicated by identifying Epromoters, a subset of promoters with enhancer function. Here, we uncovered a dual *cis*-regulatory element, “ESpromoter,” exhibiting both enhancer and silencer function as a regulator of the nearby genes *ATP2B4* and *LAX1* in single human T cells. Through an integrative approach, we pinpointed functional rs11240391, a severe malaria-risk variant that escapes detection in genome-wide association studies, challenging conventional strategies for identifying causal variants. CRISPR-modified cells demonstrated the regulatory effect of ESpromoter and rs11240391 on *LAX1* expression and T cell activation. Furthermore, our findings revealed an epistatic interaction between ESpromoter SNPs and rs11240391, impacting severe malaria susceptibility by further reducing *LAX1* expression. This groundbreaking discovery challenges the conventional enhancer-silencer dichotomy. It highlights the sophistication of transcriptional regulation and argues for an integrated approach combining genetics, epigenetics, and genomics to identify new therapeutic targets for complex diseases.

## Highlights

• Dual enhancer-silencer element (ESpromoter) in a single human cell type
• Functional SNP for severe malaria risk that escaped genome-wide association studies
• Genome editing at the SNP impacts *LAX1* expression and T cell activation
• Epistatic interaction between SNPs increases the risk of severe malaria

## Graphical abstract

**Figure:**
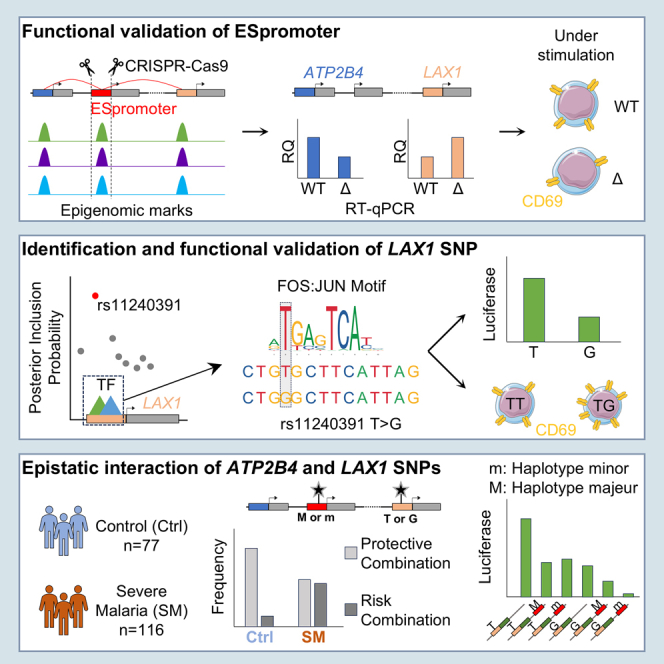

## Teaser

Adjemout and Nisar et al. identify a dual enhancer-silencer regulatory element, the “ESpromoter.” They discovered an epistatic interaction between the ESpromoter haplotype and an *LAX1* promoter variant, which escaped GWAS detection and is responsible for severe malaria susceptibility through T cell activation. They demonstrate the power of multi-omics approaches to decipher regulatory mechanisms.

## Introduction

Gene expression is tightly regulated in time and space to ensure proper gene activation. Disruptions in this process can lead to various disorders, including cancers,^1^^,^^2^; neurological^3^^,^^4^ and developmental disorders; and autoimmune, cardiovascular, and infectious diseases.^5^ Gene-expression profiling helps uncover transcriptomic signatures associated to pathological mechanisms of complex diseases.^6^^,^^7^^,^^8^^,^^9^^,^^10^^,^^11^^,^^12^^,^^13^

In humans, regulation involves proximal promoters and distal enhancers,^14^ with some promoters also acting as enhancers, termed Epromoters.^15^^,^^16^^,^^17^^,^^18^ By assessing all human core promoters of coding genes, it was shown that around 3% of promoters exhibited enhancer activity in each cell line.^15^ These Epromoters often regulate several distal genes, suggesting that they may function as regulatory hubs for the coordinated regulation of gene clusters.^16^^,^^18^ Moreover, Epromoters appear to be associated with a higher density of transcription factor (TF) binding and a higher quality of binding sites.^15^^,^^16^ This is consistent with Epromoters being preferentially associated with housekeeping^19^ and stress-response genes,^20^ including interferon-response genes. Investigating promoters, enhancers, or Epromoters is essential to understanding gene regulation, as they can affect multiple genes and influence physiological disorders. Variants within these regulatory elements can disrupt TF binding, altering gene expression and contributing to disease. Over 90% of the variants uncovered through genome-wide association studies (GWASs) are located in non-coding genome regions, frequently lacking functional annotations.^21^^,^^22^ Understanding their impact is crucial for uncovering disease mechanisms. In the case of severe malaria (SM), many loci have been found, but the causal variants and mechanisms remain unclear, highlighting the need for new approaches.

To address this challenge, we developed an innovative strategy to identify causal variants within regulatory elements associated with SM by dissecting their impact on gene expression. This work is crucial for understanding the regulatory mechanisms governing malaria susceptibility and for guiding the development of new therapies. We built a comprehensive pipeline combining genetic and epigenomic data through bioinformatics and experimental approaches. Our previous research identified an Epromoter within the *ATP2B4* locus,^17^ containing five regulatory variants (rs11240734, rs1541252, rs1541253, rs1541254, and rs1541255) associated with SM^17^^,^^23^ and in strong linkage disequilibrium (LD) with the non-functional GWAS lead single nucleotide polymorphism (SNP) (rs10900585).^24^^,^^25^^,^^26^^,^^27^^,^^28^

The current challenge is to link the *ATP2B4* Epromoter to the genes and specific cell types it affects to uncover the mechanisms driving malaria susceptibility. We identified a dual *cis*-regulatory element acting as both an enhancer (E) for *ATP2B4* and a silencer (S) for *LAX1* in the same human cell type, which we called ESpromoter. We further showed that the silencing effect on *LAX1* is modulated by a functional variant (rs11240391) in its promoter, revealing an epistatic interaction. This variant, absent from GWAS datasets due to its lack of LD with other SNPs in African populations, highlights the limitation of GWASs. Our study demonstrates that a multi-omics approach is more effective for identifying functional regulatory elements and causal variants.

## Results

### *LAX1* is a target of *ATP2B4* Epromoter

**Figure 1:**
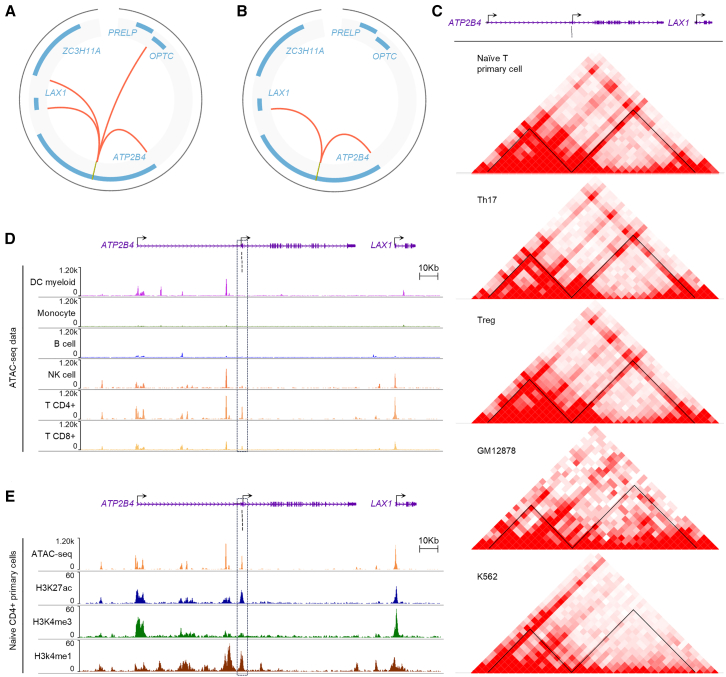
Epromoter in the *ATP2B4* locus, a potential *cis*-regulatory element of *LAX1* (A) Circos plots illustrating predictive chromatin interactions of GeneHancer regulatory elements with the *ATP2B4* Epromoter (yellow line). Predictions indicate potential interactions with multiple target promoters, including *ATP2B4*, *LAX1*, *ZC3H11A*, *PRELP*, and *OPTC*. These interactions were computationally inferred based on regulatory-element annotations. (B) Circos plots displaying significant chromatin interactions with the *ATP2B4* Epromoter (yellow line) in primary T CD4^+^ cells as measured by promoter capture Hi-C (PCHiC) data.^29^ Experimentally validated interactions confirm strong contacts with *ATP2B4* and *LAX1* promoters in this specific cell type. (C) Chromatin interaction maps reveal *ATP2B4* Epromoter interactions across multiple cell types. Hi-C interaction matrices from HiChiP data^30^ are shown for different T cell subtypes (naive T primary, Th17, and Treg cells) as well as non-T cell lines (GM12878 and K562). Strong interactions, indicated by darker shades of red, highlight the *ATP2B4* Epromoter’s contact with the *ATP2B4* and *LAX1* promoters across all tested CD4^+^ T cell lines. Black lines correspond to the five SNPs previously identified (rs11240734, rs1541252, rs1541253, rs1541254, and rs1541255). The interaction between the *ATP2B4* Epromoter and the *LAX1* promoter is consistently observed in all CD4^+^ T cell lines^30^ (naive T, Th17, and Treg), further confirming its presence in T cells. However, this interaction appears less prominent or absent in non-T cell lines. (D) WashU epigenome browser view of ATAC-seq for immune cells.^31^ The frame corresponds to the Epromoter. Black lines correspond to the five SNPs previously identified (rs11240734, rs1541252, rs1541253, rs1541254, and rs1541255). The regions corresponding to the Epromoter and the *LAX1* promoter are particularly open in CD4^+^ T cells. (E) WashU epigenome browser view of epigenomic data in naive CD4^+^ primary cells. The frame corresponds to the Epromoter. Black lines correspond to the five SNPs previously identified (rs11240734, rs1541252, rs1541253, rs1541254, and rs1541255). The presence of prominent peaks for active histone marks suggests a regulatory role for the *ATP2B4* Epromoter and *LAX1* promoter in CD4^+^ T cells.

The *ATP2B4* internal promoter, characterized as an Epromoter, may regulate distant genes. GeneHancer predictions suggest four potential targets: *ATP2B4*, *LAX1*, *ZC3H11A*, and *OPTC* (Figure 1A). Promoter capture high throughput chromosome conformation capture (Hi-C) (PCHi-C) data from CD4^+^ T cells^29^ revealed a significant T cell-specific interaction between the *ATP2B4* Epromoter and both the *LAX1* and *ATP2B4* promoters (Figures 1B and S1) but not with *ZC3H11A* or *OPTC*.

We analyzed chromatin immunoprecipitation (HiChIP) data based on H3K27ac from various cell types, including T cells.^30^ The *ATP2B4* Epromoter interacts with its *ATP2B4* promoter in all tested cell types (Figure 1C). Its interaction with the *LAX1* promoter, first seen in primary CD4^+^ T cells by PCHiC data,^29^ was also confirmed in regulatory T (Treg), T helper 17 (Th17), and naive CD4^+^ T cell lines via HiChIP^30^ (Figure 1C). This suggests a T cell-specific dual-regulatory role for the Epromoter.

### *ATP2B4* Epromoter and the *LAX1* promoter are active chromatin regions in CD4^+^ T cells

We looked at chromatin features that may be useful for identifying potential regulatory regions and their cell-specific function. Assay for transposase-accessible chromatin with high-throughput sequencing (ATAC-seq) analysis of various human immune cells^31^ confirmed cell-specific chromatin accessibility at the *ATP2B4* locus (Figure 1D). Particularly, high accessibility was observed in CD4^+^ T at the malaria-associated regulatory region and the *LAX1* promoter. These regions also showed chromatin accessibility in CD8^+^ T cells and natural killer cells (Figure 1D). H3K27Ac profiles from Roadmap data,^32^ revealed active regulatory elements at the *ATP2B4* Epromoter, containing malaria-associated SNPs and the *LAX1* promoter in CD4^+^ T cells (Figure 1E). The *ATP2B4* Epromoter showed stronger H3K4me1 than H3K4me3 mark, typical of enhancers, while the *LAX1* promoter had a dominant H3K4me3 mark, consistent with active promoters. These results confirm both as active chromatin regions in CD4^+^ T cells. Overall, the data indicate a strong correlation between DNA accessibility and histone marks, supporting T cells as a pertinent model for investigating the underlying regulatory mechanisms. Similar epigenetic profiles in Jurkat cells (Figure S2) further justified their use in downstream experiments.

### The *ATP2B4 cis*-regulatory element has a dual enhancer-silencer function in a single cell type

**Figure 2:**
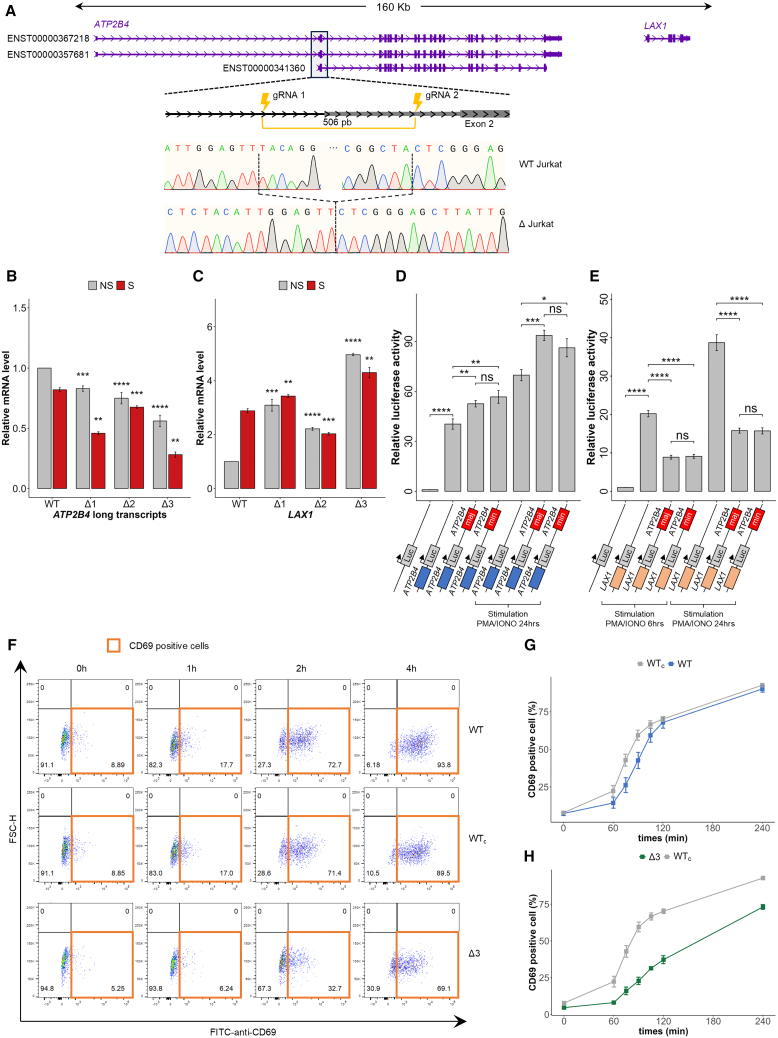
Identification of a *cis*-regulatory element with a dual enhancer-silencer function (ESpromoter) and its role in inhibiting Jurkat T cell activation (A) Generation of cell lines with a 506-bp deletion of the ESpromoter using two single guide RNAs (gRNA1 and gRNA2) flanking the regulatory region containing the five SNPs, through CRISPR-Cas9 technology. The position of the deletion is indicated relative to the long *ATP2B4* (ENST00000367218 and ENST00000367218) and short (ENST00000341360) transcripts of *ATP2B4* and the *LAX1* gene. Sanger sequencing chromatograms show the genomic sequence of the wild-type (WT) Jurkat clone, and one representative edited clone (Δ Jurkat). (B) RT-qPCR analysis of *ATP2B4* long transcript expression (ENST00000357681, ENST00000367218) on WT Jurkat cells and clones deleted for the ESpromoter (Δ1, Δ2, and Δ3) in culture without stimulation (NS, no stimulation [in gray]) or with PMA/ionomycin stimulation for 6 h (S, stimulated [in red]). Clones with a deletion had a decreased *ATP2B4* expression under both conditions. Values were generated from three independent experiments performed in triplicate. WT Jurkat NS and S are used as references. All data represent mean values ±SEM. Two-sided Student’s t tests and asterisks indicate significance (∗∗*p* < 0.01, ∗∗∗*p* < 0.001, ∗∗∗∗*p* < 0.0001). (C) RT-qPCR analysis of *LAX1* expression on WT Jurkat cells and on clones deleted for the ESpromoter (Δ1, Δ2, and Δ3) without stimulation (NS, no stimulation [in gray]) or with PMA/ionomycin stimulation for 6 h (S, stimulated [in red]). Clones with a deletion had increased *LAX1* expression under both conditions. Values were generated from three independent experiments performed in triplicate. WT Jurkat NS and S are used as references. All data represent mean values ±SEM. Two-sided Student’s t tests and asterisks indicate significance (∗∗*p* < 0.01, ∗∗∗*p* < 0.001, ∗∗∗∗*p* < 0.0001). (D) Graphs showing the relative luciferase activity under the control of *ATP2B4* promoter alone or in combination with the ESpromoter region containing either the major haplotype (maj: TCCGA) or minor haplotype (min: CTTGG) for the five SNPs (rs11240734, rs1541252, rs1541253, rs1541254, and rs1541255) without and with PMA/ionomycin stimulation. Luciferase assays confirmed the enhancer effect of the ESpromoter on the *ATP2B4* promoter independently of the haplotype. Values were generated from three independent experiments performed in triplicate. All data represent normalized mean values ±SEM. Two-sided Student’s t tests and asterisks indicate significance (ns, not significant; ∗*p* < 0.05, ∗∗*p* < 0.01, ∗∗∗*p* < 0.001, ∗∗∗∗*p* < 0.0001). (E) Graphs showing the relative luciferase activity under the control of *LAX1* promoter alone or in combination with the ESpromoter region containing either the major haplotype (maj) or minor haplotype (min) for the five SNPs (rs11240734, rs1541252, rs1541253, rs1541254, and rs1541255) under PMA/ionomycin stimulation (6 and 24 h). Luciferase assays confirmed the silencer effect of the ESpromoter on the *LAX1* promoter independently of the haplotype. Values were generated from three independent experiments performed in triplicate. All data represent normalized mean values ±SEM. Two-sided Student’s t tests and asterisks indicate significance (ns, not significant; ∗∗∗∗*p* < 0.0001). (F) T cell activation was assessed based on CD69 staining and flow cytometric gating strategy. Plots showing the results at different time points after PMA/Ionomycin stimulation of WT Jurkat cells, WT clone after CRISPR-Cas9 editing (WTc), and deleted clone for the ESpromoter (Δ3). Representative experiments on 2,000 cells according to forward scatter-horizontal (FSC-H) and anti-CD69 staining with fluorescein isothiocyanate (FITC). The number represents the percentage of cells. The orange window corresponds to CD69-positive cells. The rate of CD69-positive cells increased with stimulation time. (G) Monitoring Jurkat cell activation by anti-CD69 staining through flow cytometry after PMA/ionomycin stimulation. Values represent the average ± SEM of two independent experiments performed in duplicate, indicating the percentage of cells positive for anti-CD69 staining (acquisition of 2,000 cells per clone) for WT Jurkat, and WT clone without genomic edition after CRISPR-Cas9 (WTc). A similar percentage of CD69-positive cells was observed between WT and WTc. (H) Monitoring Jurkat cell activation by anti-CD69 staining through flow cytometry after PMA/ionomycin stimulation. Values represent the average ±SEM of two independent experiments performed in duplicate, indicating the percentage of cells positive for anti-CD69 staining (acquisition of 2,000 cells per clone) for WT clone without genomic edition after CRISPR-Cas9 (WTc) and deleted clones for the ESpromoter (Δ3). A lower number of CD69-positive cells was observed in the Δ3 clone.

We performed genome-editing experiments in the Jurkat cell line to determine the regulatory function of the *ATP2B4 cis*-regulatory element (CRE) on the target genes (*ATP2B4* and *LAX1*). Using CRISPR-Cas9, we deleted a 506-bp region containing the SNPs rs11240734, rs1541252, rs1541253, rs1541254, and rs1541255 (Figure 2A). Three biallelic deletion clones (Δ1, Δ2, Δ3) were generated, along with a wild-type (WT) control clone exposed to the CRISPR-Cas9 complex but unedited. Gene expression was assessed with and without phorbol 12-myristate 13-acetate (PMA)/ionomycin stimulation, as *LAX1*, which is involved in T cell antigen receptor (TCR) signaling, requires stimulation for induction. In the WT, *ATP2B4* long transcript levels slightly decreased upon stimulation (Figure 2B) as previously observed.^33^ Deletion of the 506-bp region in the Jurkat cells resulted a stimulation-independent decrease in the expression of *ATP2B4* long transcripts (1.8-, 1.2- and 2.9-fold decrease for Δ1, Δ2, and Δ3, respectively, in stimulated condition; *p* < 0.01) (Figure 2B). The *LAX1* gene has a PMA/ionomycin-inducible promoter whose expression increased 3-fold in WT Jurkat cells after stimulation (Figure 2C). Deletion of this region caused a significant increase in *LAX1* gene expression in Δ1, Δ2, and Δ3 clones, even in the absence of stimulation (Figure 2C).

Deletion of the *ATP2B4 cis*-regulatory element led to reduced *ATP2B4* expression and increased *LAX1* expression in Jurkat cells, indicating its dual role as an enhancer for *ATP2B4* and a silencer for *LAX1*. This supports the identification of a novel regulatory element, the ESpromoter, with both functions in the same cell type (Figure S1).

### The dual enhancer-silencer function is independent of the variants within the ESpromoter

To confirm the dual function of the *cis*-regulatory element of *ATP2B4* (ESpromoter) and assess SNPs effects on gene regulation, we performed luciferase gene reporter assays in Jurkat cells. Using a construct with the ESpromoter downstream of the luciferase reporter gene and the 780-bp *ATP2B4* promoter upstream, we confirmed strong promoter activity (40-fold increase, *p* < 0.0001) and enhancer activity of the ESpromoter (1.3-fold increase, *p* < 0.01), both with and without stimulation (Figure 2D). No significant difference was observed between the constructs with major (rs11210734T-rs1541252C-rs1541253C-rs1541254G-rs1541255A) or minor (rs11210734C-rs1541252T-rs1541253T-rs1541254C-rs1541255G) haplotype for the five SNPs located in the ESpromoter (Figure 2D). Replacing the *ATP2B4* promoter with the 810-bp *LAX1* promoter, we observed strong inducible promoter activity compared to basic vector (20-fold at 6 h, 39-fold at 24 h, *p* < 0.0001) and a consistent silencing effect by the ESpromoter (∼2.4-fold decrease, *p* < 0.0001) (Figure 2E) independent of SNP haplotypes. These results confirm the ESpromoter’s allele-independent dual enhancer-silencer activity.

### Increased expression of *LAX1* in clones lacking the ESpromoter region delayed T cell activation

Since LAX1 functions as a negative regulator of lymphocyte signaling,^34^ an increase in *LAX1* expression is expected to prevent T lymphocyte activation. To evaluate this, we measured CD69 surface expression, a marker of T cell activation, in WT and ESpromoter-deleted Jurkat clones after PMA/ionomycin stimulation. We showed that the number of CD69-positive cells was similar for WT Jurkat cells and the unedited WT clone exposed to CRISPR-Cas9 (WTc) at all stimulation times (Figures 2F and 2G). The number of CD69-positive cells increased with stimulation time for the WT, WTc, and Δ3. While CD69-positive cells increased over time for WT, WTc, and Δ3, clone Δ3 consistently showed a significantly reduced CD69 expression compared to WT and WTc (90% vs. 70% at 4 h, *p* = 0.001, paired t test) (Figures 2F and 2H).

### ESpromoter sequence is essential for normal human primary T cell activation

**Figure 3:**
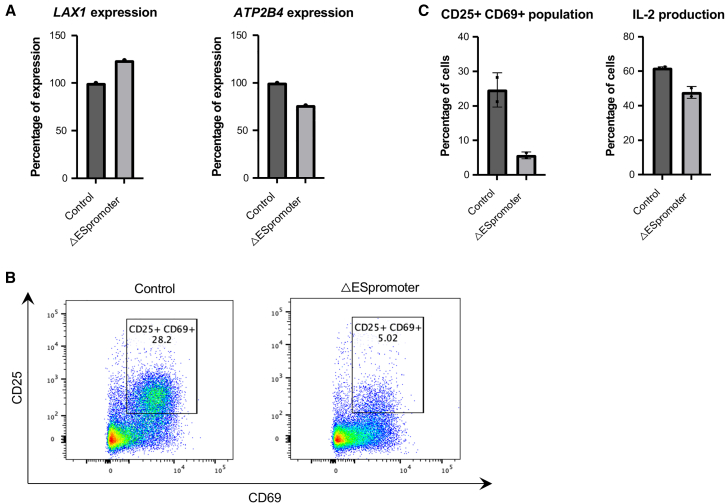
ESpromoter is essential for normal human primary T cell activation (A–C) Phenotypic analysis of naive CD4^+^ T cells, either deleted or not for ESpromoter, following 24 h activation with CD3/CD28/CD2 complexes, including a 4-h restimulation step with PMA/ionomycin/BFA; see also Figure S3. (A) Relative expression of *LAX1* (left) and *ATP2B4* (right) assessed by RT-qPCR in one donor. Deletion of the ESpromoter leads to an increase in *LAX1* expression and a decrease in *ATP2B4* expression. (B) Surface expression of CD25 and CD69 in primary control T cells and in cells deleted for the ESpromoter. Data are representative of two independent experiments. The number of CD25^+^CD69^+^ double-positive cells is shown as a percentage. (C) Quantification of CD25^+^CD69^+^ double-positive cells (left) and IL-2-producing cells (right). Data are from two independent experiments. A strong decrease in CD25^+^ CD69^+^ cells and a decrease in the percentage of IL-2-producing cells were observed after the deletion of the ESpromoter.

To investigate the functional role of ESpromoter in human primary T cells, we performed CRISPR-Cas9-mediated deletion of its sequence in purified naive CD4+ T cells (Figure S3A), followed by 24 h of TCR activation using CD3/CD28/CD2 soluble antibody complexes. To assess cytokine production, a 4-h restimulation step with PMA/ionomycin/brefeldin A (BFA) was included. The genomic deletion was validated using PCR primers, flanking the three guide RNAs (Figure S3B). Loss of ESpromoter resulted in a 24.3% increase in *LAX1* expression and a 23.5% decrease in *ATP2B4* expression compared to the control, consistent with the dual enhancer-silencer function of the ESpromoter (see STAR Methods and Figure 3A). At the phenotypic level, ESpromoter deletion led to a significant reduction in T cell activation markers, characterized by a marked decrease in the CD69^+^CD25^+^ double-positive population (mean of 24.7% in control vs. 5.7% after deletion) (Figures 3B and 3C) and impaired interleukin 2 (IL-2) secretion (mean of 62% in controls vs. 47.8% after deletion) (Figure 3C). These findings suggest that the dual enhancer-silencer function of ESpromoter is also relevant in primary T cells, playing a critical role in regulating activation.

### Discovery of the functional variant rs11240391 in the *LAX1* promoter

**Figure 4:**
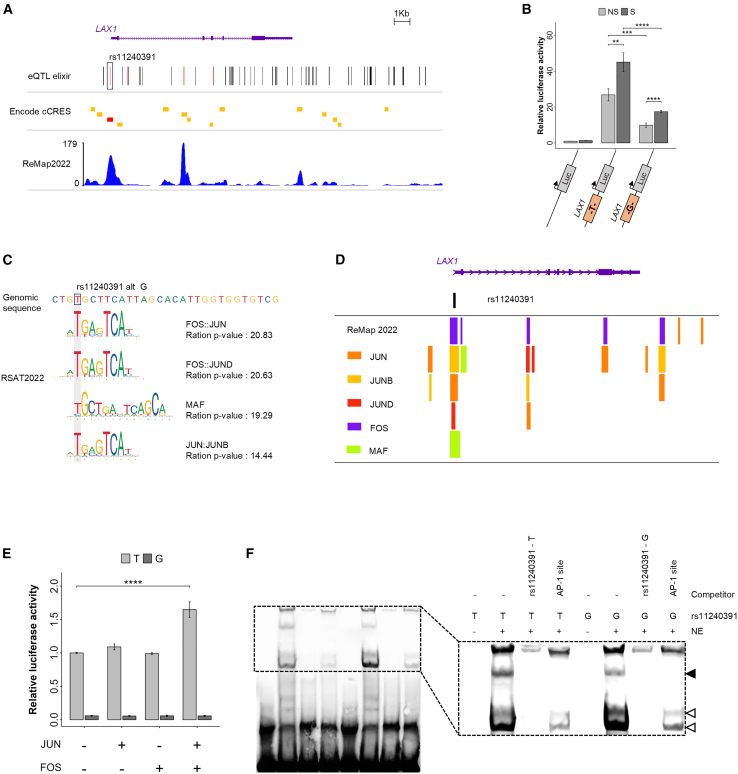
Identification of the functional variant rs11240391 corresponding to the FOS and JUN binding site (A) WashU epigenome browser view of *LAX1* region with the position of all eQTLs for *LAX1* in the ELIXIR database, those having a PIP score >0.7 are shown in red. Localization of ENCODE cCREs was shown with promoter-like elements in red and enhancer-like elements in orange. The density of ChIP-seq peaks available in ReMap2022 is also displayed. These results revealed a potentially functional SNP in the *LAX1* promoter (rs11240391). (B) Luciferase assays to assess the impact of the rs11240391 variant on *LAX1* promoter activity without stimulation (NS, no stimulation [in gray]) or with PMA/ionomycin stimulation for 6 h (S, stimulation [in black]). Values were generated from three independent experiments performed in triplicate. Relative luciferase activity was lower with the G allele than with the T allele. The graph shows the mean values ±SEM. *p* values were calculated using a two-sided Student’s t test, and asterisks indicate significance (∗∗*p* < 0.01, ∗∗∗*p* < 0.001, ∗∗∗∗*p* < 0.0001). (C) Prediction of transcription factor (TF) binding-site disruption at rs11240391 using RSAT. The genomic sequence containing the variant is displayed. The *p*-value ratio was calculated by dividing the best and worst probability of TF binding. All TFs exhibit a higher binding affinity to the T allele of SNP rs11240391. The FOS::JUN motif is the most affected by the allele change. (D) ChIP-seq peaks from ReMap2022 confirmed the binding of TFs, identified by RSAT, in the region containing rs11240391. (E) Luciferase assays to evaluate the impact of rs11240391 on the fixation of TFs by adding expression plasmid of JUN or FOS or both. The major allele T was represented in gray, and the minor allele G was represented in black. A higher relative luciferase activity was observed in the presence of the T allele, which only increased with the simultaneous addition of JUN and FOS. Values were generated in triplicate from three independent experiments. The plot shows the mean values ±SEM. *p* values were calculated using a two-sided Student’s t test, and asterisks indicate significance (∗∗∗∗*p* < 0.0001). (F) EMSA for SNP rs11240391. Nuclear extracts (NE:10 μg) from Jurkat cells stimulated for 2 h with PMA/ionomycin were incubated with biotinylated doubled-stranded oligonucleotides containing either the major T allele (rs11240391 - T) or the alternative G allele (rs11240391 - G). Two different competitions were performed either with an excess of non-biotinylated duplex identical to the biotinylated duplex or with an excess of the non-biotinylated duplex containing a specific AP-1 site. Complexes were separated on 6% nondenaturing polyacrylamide gels. Positions of specific (filled arrow) and non-specific (open arrow) AP-1 retardation are indicated. The intensity of the specific AP-1 complex band decreased in the presence of the biotinylated duplex containing the G allele.

We found no evidence of allele-specific regulation of *LAX1* by the five *ATP2B4* SNPs in the ESpromoter. We therefore investigated whether SNPs within the *LAX1* promoter, identified by Hi-C experiments as the target region of the ESpromoter (Figure 1B), could indeed functionally regulate its expression. We analyzed expression quantitative trait loci (eQTLs) using the ELIXIR eQTL catalog,^35^ revealing several SNPs with a posterior inclusion probability (PIP) score >0.2 (Figure 4A), indicating the probability of the variant being functional. Among them, rs11240391, located in the *LAX1* promoter, exhibited a high PIP score of 0.76 (*p* = 1.09 × 10^−10^) in whole-blood tissue (Figure 4A) and was also reported as an eQTL for *LAX1* in CD4^+^ T cells (PIP = 0.92) in the FIVEx database.^36^ Furthermore, the ENCODE data confirm that the *LAX1* promoter region corresponds to a candidate *cis*-regulatory element (cCRE) (Figure 4A), where numerous chromatin immunoprecipitation sequencing (ChIP-seq) peaks for TFs were identified using ReMap2022^37^ (Figure 4A). To study the effect of rs11240391 on *LAX1* expression, we cloned the *LAX1* promoter in Jurkat cells, with each allele into a luciferase reporter construct, and we measured relative expression in the presence or absence of PMA/ionomycin stimulation. Upon stimulation, luciferase expression increased for both the T and G alleles (Figure 4B). However, the minor G allele at rs11240391 showed reduced expression compared to the major T allele under both conditions (*p* < 0.001) (Figure 4B), confirming the functional role of this SNP (Figure S1).

### The regulatory function of the rs11240391 SNP was mediated by the FOS and JUN TFs

To uncover the regulatory mechanism and determine whether TF binding may be different between rs11240391 alleles, we used the RSAT tool. Hence, we identified *in silico* several factors whose binding seemed to be affected by this SNP (Figure 4C), suggesting preferential binding of these factors on the T allele. The best *p*-value ratio was obtained for FOS::JUN (*p* = 20.83). ChIP-seq data from ReMap 2022^37^ indicated the binding of the TFs JUN, JUNB, JUND, FOS, and MAF to the *LAX1* promoter (Figure 4D). Therefore, we next focused on these two TFs and assessed by luciferase assay in Jurkat cells whether the rs11240391 variant of the *LAX1* promoter could be a potential target of FOS and JUN. As shown in Figure 4E, in the presence of plasmids expressing the FOS and JUN proteins, relative luciferase activity was significantly increased (*p* < 0.0001, 1.69-fold increase) only in the presence of the T allele of the rs11240391 variant. A clear potentialization of the activation effects can be observed in the presence of FOS and JUN, allowing the formation of the activator protein 1 (AP-1) dimer, specifically on the T allele, as predicted by RSAT (Figure 4C). To confirm allele-specific protein binding, we performed an electrophoretic mobility shift assay (EMSA) using 30-bp biotinylated DNA fragments centered upon each allele of rs11240391 together with nuclear extracts from the Jurkat cell line stimulated for 2 h with PMA/ionomycin. The SNP rs11240391 showed unbalanced binding between alleles, revealing three complexes, one showing higher intensity and two showing lower intensity for the major T allele compared with the G allele (Figure 4F). The complex corresponding to the upper band disappeared when we used an excess of unlabeled oligonucleotides containing the AP-1 sequence^38^ (Figure 4F), supporting that the differences in retardation reflect differences in protein binding to the AP-1 site. Therefore, this specific AP-1 shift confirms the preferential binding of AP-1 to the T allele of rs11240391, as expected based on bioinformatics predictions and luciferase assays (Figure 4F).

### Genetic editing of rs11240391 allele confirms its regulatory impact on *LAX1* expression

**Figure 5:**
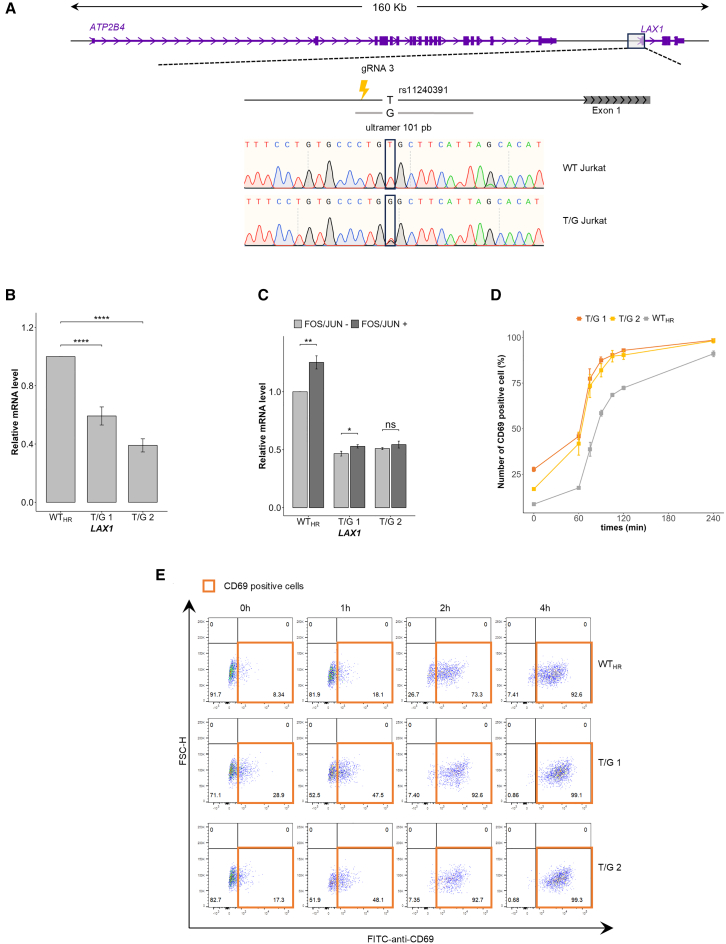
Lower *LAX1* expression in heterozygous T/G Jurkat clones is associated with higher T cell activation (A) Generation of cell lines with a modified rs11240391 variant allele by HR using a 101-bp ultramer, a single guide RNA (gRNA3), and CRISPR-Cas9 technology. Sanger sequencing chromatograms show the genomic sequence of the wild-type (WT) Jurkat clone and of the clone in which a T allele has been replaced by a G allele (T/G Jurkat). (B) RT-qPCR analysis of *LAX1* gene expression on WT clone after HR experiment (WT_HR_) and two heterozygous clones (T/G 1 and T/G 2) for the rs11240391 SNP. Values were generated in triplicate from three independent experiments. The plot shows the mean values ±SEM. *p* values were calculated using a two-sided Student’s t test, and asterisks indicate significance (∗∗∗∗*p* < 0.0001). (C) RT-qPCR analysis of *LAX1* gene expression in WT Jurkat cells (WT_HR_) and T/G clone heterozygotes (T/G 1 and T/G 2) for the SNPs rs11240391, untransfected (gray) or transfected (black) with both expression plasmids for FOS and JUN. Values were generated in triplicate from three independent experiments. The plot shows the mean values ±SEM. *p* values were calculated using a two-sided Student’s t test, and asterisks indicate significance (ns, not significant; ∗*p* < 0.05, ∗∗*p* < 0.01). (D) Monitoring of Jurkat cell activation by anti-CD69 staining through flow cytometry after PMA/ionomycin stimulation. The values represent the average ± SEM of two independent experiments performed in duplicate, indicating the percentage of cells positive for anti-CD69 staining (acquisition of 2,000 cells per clone). The comparison was carried out between a WT clone after an HR experiment (WT_HR_) and two heterozygous clones (T/G 1 and T/G 2) for the rs11240391 SNP. The percentage of CD69^+^ cells was higher in heterozygous clones than in WT_HR_. (E) T cell activation in Jurkat clones was assessed based on CD69 staining and flow cytometric gating strategy. Plots showing the results at different time points after PMA/Ionomycin stimulation of WT clone after CRISPR-Cas9 editing (WT_HR_) and heterozygous clones (T/G 1 and T/G 2) for the rs11240391 SNP. Representative experiments were conducted on 2,000 cells of each type based on forward scatter-horizontal (FSC-H) and anti-CD69 staining using fluorescein isothiocyanate (FITC). The number represents the percentage of cells. The orange window corresponds to CD69-positive cells. A higher percentage of CD69-positive cells was observed for T/G 1 and T/G 2 clones.

We performed homologous recombination (HR) with CRISPR-Cas9 genome editing to replace the T with the G allele of rs11240391 in WT Jurkat cells (Figure 5A). *LAX1* quantification was performed by RT-qPCR, showing a significant decrease of the *LAX1* transcript in both heterozygous clones compared with the WT cells unmodified after CRISPR-Cas9 (WT_HR_) (*p* < 0.001, 2.12-fold decrease) (Figure 5B).

We then assessed the effect of the addition of FOS and JUN proteins in cultured WT cells or heterozygous clones. As expected, a significant increase in *LAX1* expression was observed in WT_HR_ (T/T) cells (*p* < 0.0001, 1.65-fold increase) when the two TFs were added together (Figure 5C). Only a slight increase in *LAX1* was observed in the presence of FOS and JUN in the T/G clones compared to the WT_HR_ (T/T) clone. This result is consistent with a lower binding of FOS and JUN to the G allele of rs11240391, as predicted by the RSAT tool.

### Heterozygous rs11240391 clones display hyperactivation of TCR signaling

Based on the biological function of the *LAX1* gene, we hypothesized that a decrease in *LAX1* expression, as demonstrated in rs11240391 heterozygous clones, may increase the activation of TCR signaling. To confirm this hypothesis, we quantified the CD69 marker on the cell surface by flow cytometry before and at several points after PMA/ionomycin stimulation for WT_HR_ cells and the two T/G clones (Figures 5D and 5E). The number of CD69-positive cells was higher in the two heterozygous clones even before stimulation (28.9% and 17.1%) (Figures 5D and 5E) compared to WT cells (8.3%), suggesting basal hyperactivation consistent with reduced *LAX1* expression. The number of CD69-positive cells increased with the duration of stimulation for all three clones, with higher numbers in the T/G clones than in the T/T clone (*p* = 0.0003 and *p* = 0.0008 when WT_HR_ was compared to T/C_1_ and T/C_2,_ respectively), with almost 100% of positive cells at 4 h of stimulation for the heterozygous clones. These data demonstrate that rs11240391, a regulatory variant of *LAX1*, affects T cell activation (Figure S1).

### Association of SM with rs11240391 that was not detected in GWAS

**Figure 6:**
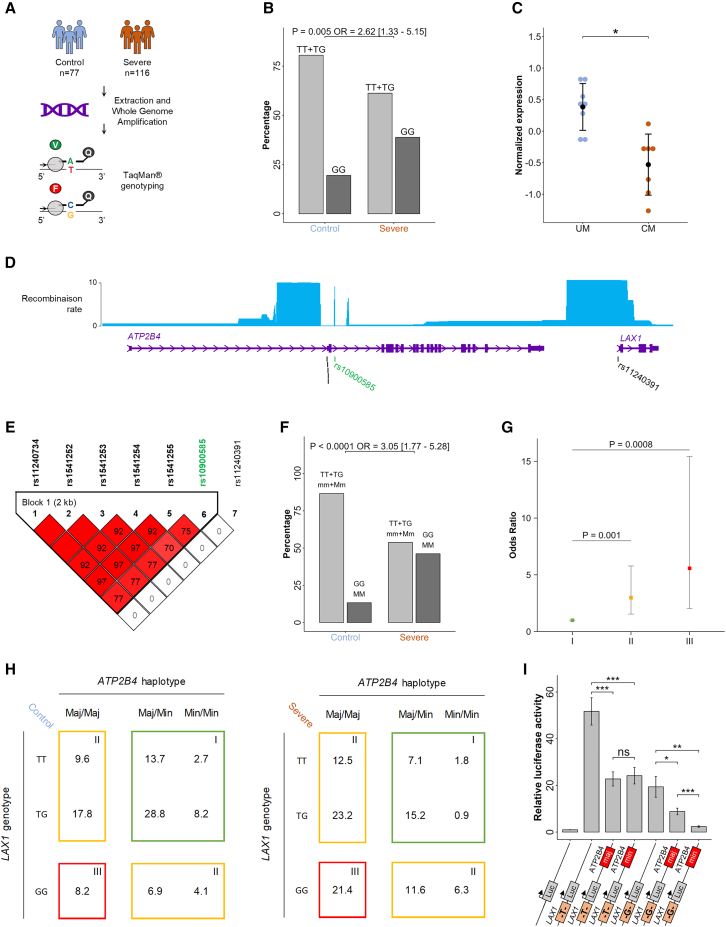
Epistatic interaction between *ATP2B4* and *LAX1* genetic variants associated with differential risk of SM through regulation of *LAX1* expression (A) Design for Senegalese cohort recruitment, DNA amplification, and genotyping of rs11240391 using TaqMan assays; see also Table S1. (B) Association of rs11240391 with severe malaria (SM). The graph shows the percentage of the GG risk genotype (black) versus heterozygous TG and major homozygous TT (gray) in the control and SM groups. *p* values were calculated using logistic regression analyses and the graph displays the odds ratio (OR) with its 95% confidence interval. GG individuals have a 2.62 risk of developing SM. (C) Normalized *LAX1* expression obtained by microarray in children with uncomplicated malaria (UM) and cerebral malaria (CM). Lower expression of *LAX1* is observed in CM. (D) WashU epigenome browser view of recombination rate of Yoruba in Ibadan, Nigeria (YRI). Black lines correspond to the five SNPs previously identified (rs11240734, rs1541252, rs1541253, rs1541254, and rs1541255) and the SNP in the *LAX1* promoter (rs11240391). The green line corresponds to the tagSNP (rs10900585) previously associated with SM in GWAS analyses. The *ATP2B4* SNPs and the *LAX1* SNP rs11240391 are in two different recombination blocks. (E) Linkage disequilibrium (LD) between different SNPs of the *ATP2B4* and *LAX1* genes in our Senegalese cohort. LD is expressed as r^2^ multiplied by 100. SNPs with an r^2^ > 0.6 are considered in LD and are colored in red. No LD was observed between the *ATP2B4* SNPs and the SNP *LAX1* rs11240391. (F) Epistatic interaction between the haplotype of five SNPs and rs11240391 computed by logistic regression. The plot shows the percentage of individuals carrying either both protective haplotype/genotype (mm + Mm/TT + TG) (m, minor haplotype; M, major haplotype) or both risk haplotype/genotype (MM/GG). Individuals carrying the GG genotype and the MM haplotype are at increased risk of SM. *p* values were calculated using logistic regression analyses and the graph displays the OR with a 95% confidence interval; see also Table S2. (G) SM risk associated with genotype-combination groups. The colored dots represent OR, and the bars represent 95% confidence intervals. Group I corresponded to the baseline SM risk (OR = 1). Group II had a moderate SM risk (OR = 2.99, 95% CI [1.54–5.78], *p* = 0.001); the risk was increased in group III (OR = 5.57, 95% CI [2.01–15.42], *p* = 0.0008). (H) Genotype combinations were identified by conditional logistic regression due to the interaction between *ATP2B4* and *LAX1* variants. Group I included individuals with at least one minor *ATP2B4* haplotype copy and at least one major T allele copy for *LAX1* (25% of SM and 53.4% of Ctrl). Group II included individuals homozygous for both major *ATP2B4* haplotype and major T allele *LAX1* as well as individuals homozygous either for major *ATP2B4* haplotypes or minor G allele of *LAX1* (53.6% of SM and 38.4% of Ctrl). Group III included individuals homozygous for both the major *ATP2B4* haplotype and for the minor G allele of *LAX1* (21.4% of SM and 8.2% of Ctrl). (I) Luciferase assays to functionally assess the epistatic effect between the haplotype, including the five SNPs in the ESpromoter, and the *LAX1* promoter SNP rs11240391. Graphs showing the relative luciferase activity under the control of *LAX1* promoter with T or G allele at rs11210391 alone or in combination with the ESpromoter containing either the major haplotype (maj: TCCGA) or minor haplotype (min: CTTGG) for the five SNPs (rs11240734, rs1541252, rs1541253, rs1541254, and rs1541255). Values were generated in triplicate from three independent experiments. The plot shows the mean values ±SEM. *p* values were calculated using a two-sided Student’s t test, and asterisks indicate significance (ns, not significant; ∗*p* < 0.05, ∗∗*p* < 0.01, ∗∗∗*p* < 0.001).

Since T cell immunity plays a central role in protection against pathogens,^39^^,^^40^^,^^41^ we hypothesize that the rs11240391 variant in the *LAX1* promoter might also be associated with SM. We found that the GG genotype was associated with an increased risk of SM in a Senegalese population (odds ratio [OR] = 2.6, *p* = 0.005) (Figures 6A, 6B and S1), with 39% and 19% in cases and controls (Ctrl), respectively. This result suggests that a diminished *LAX1* expression, leading to T cell hyperactivation, may predispose individuals to disease. To assess whether *LAX1* expression might be involved in susceptibility to SM, we re-analyzed previously published expression data by comparing the transcriptome of peripheral blood mononuclear cells (PBMCs) from children with cerebral malaria (one form of SM) and uncomplicated malaria.^6^^,^^7^ We observed that cerebral malaria was associated with significantly reducing *LAX1* expression (*p* = 0.025, 1.85-fold decrease) (Figure 6C).

The association signal for rs11240391, identified here, was not detected in previous GWASs, as this SNP is not included among the genotyped variants and is in very low LD with all other SNPs (r^2^ < 0.4, except for rs6680886 having an r^2^ of 0.56) in African populations (Figure S4). The LD between rs11240391 and the TagSNP rs10900585 associated with SM by GWAS was estimated to be r^2^ = 0.0015 in African populations using the web-based LDlink.^42^ Consistently, the SNP rs11240391 is in a region with a high recombination rate (data from Yoruba in Ibadan, Nigeria), which favors the absence of polymorphisms in LD with it (Figure 6D). We then confirmed the absence of LD in our Senegalese population between rs11240391 and the GWAS lead SNP (rs10900585), as well as with the five SNPs within the *ATP2B4* ESpromoter (Figure 6E).

### Epistatic interaction between rs11240391 and SNPs at ESpromoter

Considering the established involvement of the ESpromoter in *LAX1* gene regulation and its physical interaction with the *LAX1* promoter, we explored potential epistatic interactions between rs11240391 and the five SNPs of the ESpromoter. Individuals carrying both the GG risk genotype at rs11240391 and the *ATP2B4* risk haplotype (major haplotype) had a higher risk of developing SM with 21% and 8% in SM and Ctrl, respectively (OR = 3.05, *p* < 0.0001) (Figures 6F and S1; Table S2), suggesting an epistatic interaction between them. The logistic regression analysis highlighted a strong interaction between the *ATP2B4* and *LAX1* variants (*p* = 0.000118), and the best-fit model reveals three genetic combinations, each associated with different levels of SM risk (Figure 6G). Individuals having at least one minor *ATP2B4* haplotype copy and at least one T allele copy for *LAX1* were at the lowest risk and were thus the baseline for OR calculation (group I, 25% of SM and 53.4% of Ctrl) (Figures 6G and 6H). The highest risk (*p* = 0.0008; OR = 5.57; 95% confidence interval [CI], 2.01–15.42) was seen in individuals homozygous for both the major *ATP2B4* haplotype and the minor G allele of *LAX1* (group III: 21.4% SM, 8.2% Ctrl) (Figures 6G and 6H). Intermediate risk (OR = 2.99, *p* = 0.001; 95% CI, 1.54–2.78) was observed for the remaining individuals (group II: 53.6% SM, 38.4% Ctrl; includes only 10 individuals homozygous for both minor *ATP2B4* haplotype and minor G allele of *LAX1*) (Figures 6G and 6H). We carried out haplotype analysis, combining rs11240734, rs1541252, rs1541253, rs1541254, rs1541255, and rs11240391, revealing four haplotypes showing different frequencies between cases and Ctrl individuals: (1) major *ATP2B4* haplotype with *LAX1* rs11240391 minor G allele (0.42 for SM versus 0.28 for Ctrl), (2) major *ATP2B4* haplotype with *LAX1* rs11240391 major T allele (0.31 SM, 0.32 Ctrl), (3) minor *ATP2B4* haplotype with *LAX1* rs11240391 minor G allele (0.17 SM, 0.20 Ctrl), and (4) minor *ATP2B4* haplotype with *LAX1* rs11240391 major T allele (0.10 SM, 0.20 Ctrl). Haplotype (1) was associated with increased risk of SM (*p* = 0.004), whereas haplotype (4) was associated with protection (*p* = 0.003). These results support an epistatic interaction between *ATP2B4* haplotype and *LAX1* rs11240391 variant. We assessed the functional relevance of this epistatic interaction by luciferase reporter assays. We validated the haplotype-independent silencer impact of the ESpromoter on the *LAX1* promoter with *LAX1* rs11240391 major T allele (*p* < 0.001, with a 2.27- and 2.13-fold decrease with the major haplotype or the minor haplotype, respectively). However, with *LAX1* rs11240391 minor G allele, the ESpromoter silencer effect was haplotype dependent, showing a stronger repression with the minor *ATP2B4* haplotype (*p* < 0.01; 2.19-fold vs. 8.18-fold decrease) (Figure 6I).

We demonstrated an epistatic interaction, as the ESpromoter’s regulatory effect depends on the rs11240391 allele, revealing a functional, synergistic effect between them.

## Discussion

To elucidate the regulatory dynamics of the *ATP2B4* locus and its role in SM, we used an integrative approach combining genetics, epigenomics, and CRISPR-Cas9 genome editing (Figure S1). This led to the discovery of an ESpromoter, a *cis*-regulatory element with both enhancer and silencer functions in a single cell type. We also identified a functional SNP (rs11240391) associated to SM and revealed its epistatic interaction with ESpromoter variants. Our results show that this multi-layered strategy effectively identifies regulatory elements and causal variants, addressing the limitations of GWASs used alone.

**Figure 7:**
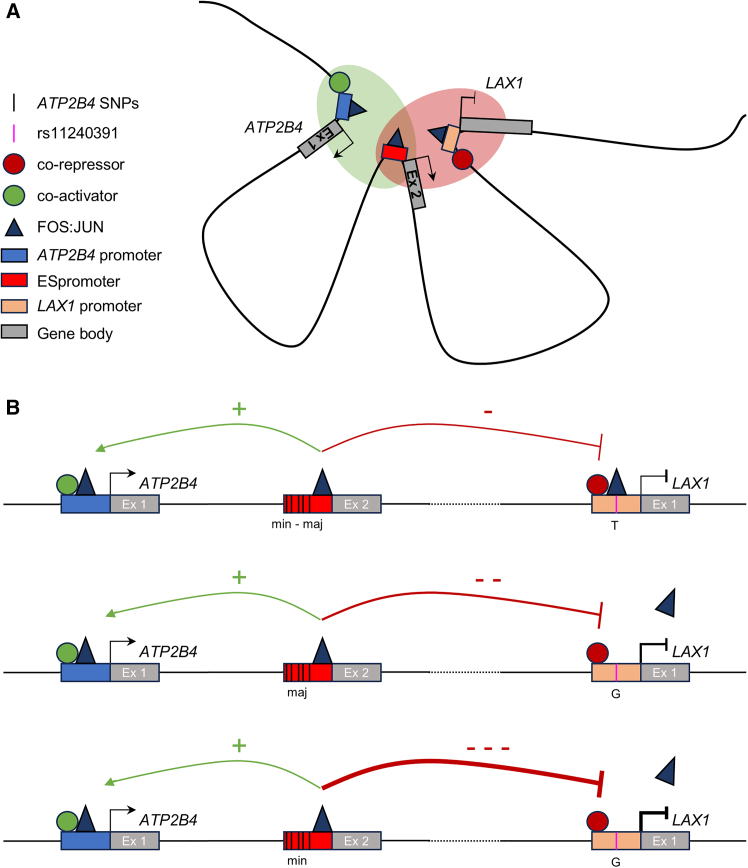
Model of the dual enhancer-silencer function of ESpromoter, epistatic interaction, and gene regulation (A) Chromatin interactions place promoters in close physical proximity, facilitating the recruitment of TFs needed to activate or repress transcription of their associated genes. The presence of an enhancer-silencer promoter (ESpromoter) within a regulated gene cluster could facilitate the assembly or maintenance of the TFs and cofactors by tightening the promoter-promoter interactions or by providing specific transcriptional regulators required for neighboring gene regulation. (B) Expression of the long transcripts *ATP2B4* and *LAX1* is regulated by a dual enhancer-silencer regulatory element (ESpromoter) in the same cell line through genetic variants associated with SM. ESpromoter functions as an enhancer for the long *ATP2B4* transcripts independently of the haplotype of the five SNPs it contains (min, minor haplotype or Maj, major haplotype) while it functions as a silencer for *LAX1* gene with an allele-specific intensity. The five SNPs within the ESpromoter act synergistically with rs11240391 to inhibit *LAX1* gene expression. This epistatic interaction results in a stronger silencing effect in the presence of the G allele of rs11240391, for which no FOS::JUN binding is possible. Unidentified co-activators and co-repressors are also thought to modulate the activating or repressing effect on *ATP2B4* and *LAX1*, respectively.

Enhancers activate gene expression in a tissue-specific manner, while silencers repress transcription to prevent inappropriate activation.^43^ Some elements can act as enhancer or silencer, depending on the cellular context, in *Drosophila*,^44^^,^^45^^,^^46^ mice,^47^ and human.^48^ Recent studies suggest that promoters can have silencer-^49^^,^^50^ or enhancer-like function.^15^^,^^16^^,^^17^^,^^18^ Our results challenge the current view of enhancers and silencers as two distinct elements, suggesting that dual-function CREs within the same cell type represent a key, yet underexplored, layer of transcriptional regulation. The exact mechanism underlying dual elements in the same cell type remains unknown. However, evidence supports a model where the ESpromoter forms direct 3D contacts with target gene promoters ^29^, likely mediated by TFs and cofactors (Figure 7). As hubs for TF recruitment through short DNA motifs, *cis*-regulatory elements coordinate complex spatiotemporal gene regulation.^51^

We demonstrated that *LAX1* expression depends on FOS and JUN binding at its promoter, effective only with the T allele at rs11240391. AP-1 regulates gene expression in response to stimuli, including cytokines and infections, and facilitates chromatin remodeling. Interestingly, AP-1 also binds to the ESpromoter region (Figure S5) independently of the haplotype and to the *ATP2B4* promoter in which no common variants were identified. However, its role in the dual enhancer-silencer function of the ESpromoter remains unclear. Specific TFs can act as activators or repressors depending on context, influencing transcription and chromatin structure at crucial loci.^52^^,^^53^^,^^54^

*Cis*-regulatory elements often contain genetic variants, adding further complexity to their genomic function. With up to 90% of GWAS loci containing non-coding variants, identifying disease-causing regulatory elements remains a major challenge. We identified a regulatory SNP, rs11240391, in the *LAX1* promoter associated with SM. This SNP was undetectable by conventional GWAS methods due to its non-inclusion as a TagSNP and lack of LD with other SNPs in African populations. While GWASs localize risk loci, they often fail to pinpoint causal SNPs, highlighting the need for integrative strategies like ours. Furthermore, common non-coding variants, which often have low individual impact on gene expression, likely affect disease risk through modest phenotypic effects. We, therefore, propose that functional assessment of several variants simultaneously, to assess their epistatic interaction, is crucial for understanding their combined impact on disease.^17^^,^^23^ We found that regulatory function of the ESpromoter is independent of the *ATP2B4* haplotype in the presence of the major T allele at rs11240391 in Jurkat cells. However, the repressive function is stronger with the minor G allele for rs11240391, suggesting an epistatic interaction between these SNPs. This genetic interaction correlates with a significantly increased risk of SM in the Senegalese population. Dual-function elements like the ESpromoter may influence neighboring genes from the same pathway, amplifying their biological impact.

Notably, the ESpromoter simultaneously activates *ATP2B4* and represses *LAX1*, both critical for T cell activation.^33^^,^^34^ PMCA4b (plasma membrane Ca^2+^ ATPase), the main *ATP2B4* isoform in CD4^+^ T cells^33^ and Jurkat cells,^55^ controls intracellular calcium. Downregulation of PMCA4b reduced effector cells and increases naive cells,^33^ supporting that the dual *cis*-regulatory element may play a key role in immune response. We propose a biphasic^33^ model for PMCA4 expression: early repression by YY1 during calcium rise followed by activation to prevent overload and support T cell survival. Moreover, CD147 interacts with PMCA4 to bypass TCR signaling and inhibit IL-2 expression,^56^ while the LAX adaptor protein, a LAT-like molecule identified in T cells,^57^ negatively regulates the TCR signaling pathway.^34^ ESpromoter deletion increases *LAX1* and reduces *ATP2B4*, impairing T cell activation. Jurkat clones heterozygous for rs11240391 show *LAX1* downregulation and hyper-responsiveness to TCR-mediated stimulation like LAX-deficient mice,^34^ which also show enhanced calcium flux. Overall, our data suggest that PMCA4 and LAX1 play a central role in calcium homeostasis and CD4^+^ T cell activation, integrating positive and negative mechanisms to balance TCR signaling.

ESpromoters may impact various traits by regulating nearby and distant genes,^18^ even across different pathways, and alterations in these regulatory elements may lead to numerous human diseases.^58^ Variants in the ESpromoter and *LAX1* promoter likely have pleiotropic effects by affecting T cell function in adaptive immunity. The minor allelic frequency of rs11240391 is higher in Africa than in other populations, indicating that, while it is a risk factor for malaria, it may offer a protective advantage in the context of other endemic diseases. In other populations, the minor allele frequency exceeds 17%; therefore, we can speculate that this variant may be associated with immune-related diseases worldwide. The pleiotropic role of non-coding variants has recently been pointed up by the identification of a genetic variant associated with five vascular diseases.^59^ Finally, we have highlighted another layer of complexity by demonstrating an epistatic interaction between these genetic variants, which increases SM risk, by deregulating *LAX1* and hyper-activating T cells. Surprisingly, individuals with the combination of minor *ATP2B4* haplotype and minor *LAX1* allele associated with the lowest *LAX1* level are not at a higher risk of SM. We have shown that these individuals have an intermediate risk of disease and propose that this is due to the protective effect of minor *ATP2B4* haplotype in other cell types, as previously demonstrated.^17^ Understanding epistasis is key to unraveling the complexity of genetic combinations and their effect on complex diseases.

Although 3D interaction between the ESpromoter and gene promoters in CD8^+^ T cells remains unproved, open chromatin at *ATP2B4*, *LAX1* promoters, and ESpromoter suggests a similar regulatory mechanism. *LAX1* deregulation and disrupted Ca^2+^ signaling may drive CD8^+^ T cell hyperactivation, contributing to SM and particularly cerebral malaria. Immune hyperactivation may also underlie autoimmune and allergic diseases. Studying genetic effects on T cell function could reveal therapeutic targets for malaria and other immune conditions, highlighting the pleiotropic impact of variants.

### Limitations of the study

Although our study provides valuable insights, some limitations should be considered. We lack direct functional validation of the ESpromoter’s repressive mechanisms, which appear independent of H3K9me3/H3K27me3 marks in Jurkat cells, aligning with studies on silencers without these modifications. Further work is needed to identify co-repressors required for the silencer-mediated activity of the ESpromoter. We proposed AP-1 as a potential candidate for this role, which could be tested through AP-1 depletion via small interfering RNA (siRNA). We were unable to generate GG homozygous clones for rs11240391, so future base or prime editing could be performed to further explore the allele-specific effects of rs11240391 on *LAX1* expression. A larger African cohort is required to achieve sufficient statistical power to replicate the epistatic interaction between *ATP2B4* and *LAX1* genetic variants due to low variant frequencies in non-African population. Nevertheless, our study has uncovered a dual *cis*-regulatory element that may be widespread in the genome and implicated in various diseases.

## Resource availability

### Lead contact

Further information and requests for resources and reagents should be directed to and will be fulfilled by the lead contact, Sandrine Marquet (sandrine.marquet@univ-amu.fr).

### Materials availability

The materials that support the findings of this study are available from the corresponding author upon reasonable request. Please contact the lead contact, Sandrine Marquet (sandrine.marquet@univ-amu.fr), for additional information.

### Data and code availability

This paper did not generate any unique datasets or code. This paper analyzes existing, publicly available data. These accession numbers for the datasets are listed in the key resources table.

## Acknowledgments

This work was supported by the (CEA-SAMEF) at 10.13039/501100005757UCAD (to B.M.), the Pasteur Institutes in Dakar and Paris (to A.T.), the French Embassy in Dakar, and recurrent funding from 10.13039/501100001677Institut National de la Santé et de la Recherche Médicale (10.13039/501100001677Inserm) and 10.13039/100007586Aix-Marseille University. This work was also supported by the French Agency for Research (10.13039/501100001665Agence Nationale de la Recherche [ANR]) ANR-22-CE15-0025-01 “MADBIO” (to S.M.) and ANR-23-CE15-0003 “RNAMethylTcell” (to V.A.). M.A. and S.N. were supported by a PhD fellowship from the French Ministry of Research and the Higher Education Commission (HEC) from Pakistan, respectively. This project was carried out in the framework of the INSERM GOLD Cross-Cutting program (P.R. and S.M.). We thank the members of TAGC, Lydie Pradel and Nathalie Arquier, for their helpful discussions and Pauline Andrieux for her advice on CRISPR technology. Selected artwork shown in the graphical abstract was used from or adapted from pictures provided by Servier Medical Art (Servier; https://smart.servier.com/), licensed under a Creative Commons Attribution 4.0 Unported License. We thank all patients who participated in the study, the Dakar main hospital, and Tambacounda regional hospital for the recruitment of biological samples. We are grateful to Claire Detraves for technical help and to R. Romieu-Mourez and P.-E. Paulet from the human immune monitoring platform of Infinity-INSERM UMR1291 for technical assistance and the supervision of the PBMC biobank from healthy adult subjects. We also thank the Marseille-Luminy cell biology platform for the management of cell culture.

## Author contributions

Conceptualization, S.M.; methodology, M.A., S.N., A.E., R.T., M.T., H.T.N.H., I.M., V.A., and S.M.; investigation, S.S., P.R., and S.M.; Senegalese cohort, A.T., B.M., A.D., and P.R.; writing – original draft, M.A. and S.M.; writing – review & editing, M.A., S.S., P.R., V.A., and S.M.; funding acquisition, A.T., A.D., P.R., V.A., and S.M.; supervision, S.M.

## Declaration of interests

The authors declare no competing interests.

## STAR★Methods

### Key resources table

**Table:** 

REAGENT or RESOURCE	SOURCE	IDENTIFIER
**Antibodies**		

Mouse monoclonal FITC anti-human CD69	BioLegend	Cat#310904; RRID: AB_314839
BD Horizon™ Mouse anti-human CD3-BV510	BD Biosciences	Cat#563247; RRID: AB_2738093
CD4 Monoclonal antibody (RPA-TA) - FITC	eBioscience	Cat#17-0049-41; RRID: AB_1272048
BD Pharmingen Mouse anti human CD45RA – PE-Cy™	BD Biosciences	Cat#561216; RRID: AB_10611721
ImmunoCult™ Human CD3/CD28/CD2 T cell Activator	STEMCELL	Cat#10990
Brilliant Violet 711™ anti-human CD69 Antibody	BioLegend	Cat#310944; RRID: AB_2566466
PE/Cyanine7 anti-human CD25 Antibody	BioLegend	Cat#302612; RRID: AB_314281
BD Horizon™ BV750 Rat Anti-Human IL-2	BD Biosciences	Cat#554563; RRID: AB_398570

**Bacterial and virus strains**		

NEB 5-alpha competent E. coli (High efficiency)	New England Biolabs	Cat#C29871
Invitrogen™ E. coli chemically competent One Shot™ TOP10	ThermoFisher	Cat#C404003

**Biological samples**		

Severe malaria and control samples from Senegal	This paper (Table S1)	N/A
Cerebral malaria and uncomplicated malaria children from Mali	Patients Section (Cabantous et al.)^6^	https://doi.org/10.1093/infdis/jix359.
Blood Sample	EFS (DC-2015-2488)	N/A

**Chemicals, peptides, and recombinant proteins**		

Phorbol 12-myristate 13-acetate	Sigma-Aldrich	Cat#P1585
Ionomycin	Sigma-Aldrich	Cat#13909
Brefeldin A	Sigma-Aldrich	Cat#B7651-5MG
Alt-R™-s.p Hifi Cas9 Nuclease V3	Integrated DNA technologies	Cat#1081060
Alt-R® Cas9 Electroporation Enhancer 2 nM	Integrated DNA technologies	Cat#1075915
Alt-R® Cas9 Electroporation Enhancer 10 nM	Integrated DNA technologies	Cat#1075916
Alt-R™-s.p Cas9 Nuclease V3	Integrated DNA technologies	Cat#1081059
Alt-R® HDR Enhancer V2	Integrated DNA technologies	Cat#10007910
DPBS	ThermoFisher	Cat#14190094
DMSO	Sigma-Aldrich	Cat#D4540
FBS	ThermoFisher	Cat#A5256701
ImmunoCult™-XF T cell Expansion Medium	STEMCELL	Cat#10981
eFluor 506	ThermoFisher	Cat#65-0866-18

**Critical commercial assays**		

Master Mix PCR Power SYBR™ Green	Applied Biosystems	Cat#4367659
QIAamp DNA mini kit	QIAGEN	Cat#51306
Q5 Site-Directed Mutagenesis Kit	New England Biolabs	Cat#E0554S
Phire Tissue Direct PCR Master Mix	ThermoFisher	Cat#F170L
QIAquick PCR Purification Kit	QIAGEN	Cat#28106
Dual-Luciferase kit	Promega	Cat#E1910
RNeasy Plus mini kit	QIAGEN	Cat#74136
TaqMan™ Genotyping Master Mix	Applied Biosystems	Cat#4371357
Superscript VILO Master Mix	Invitrogen	Cat#11755-050
EasySep™ Human Naive CD4^+^ T cell Isolation kit II	STEMCELL	Cat#19555
P2 Primary Cell 4D-Nucleofector X kit S	Lonza Bioscience	Cat#V4XP-2024
DNeasy Blood and Tissue Kits for DNA Isolation	QIAGEN	Cat#ID69504
NucleoSpin® RNA XS kit	MACHEREY-NAGEL	Cat#740990.50
SensiFAST cDNA Synthesis Kit	Ozyme	Cat#BIO-65053
SsoAdvanced Universal SYBR® Green Supermix	BioRad	Cat#1725017
Cytofix/Cytoperm Fixation/Permeabilization kit	BD Biosciences	Cat#554714
Nuclear extract kit	Active Motif	Cat#40010
Bradford protein assay	Bio-Rad	Cat#5000006
GelshiftTM Chemiluminescent EMSA Kit	Active Motif	Cat#37341
6% polyacrylamide Novex™ DNA Retardation Gels	ThermoFisher	Cat#EC63652BOX

**Deposited data**		

Physical chromatin interaction prediction	Genehancer (Fishilevich et al.)^61^	https://hgdownload.soe.ucsc.edu/gbdb/hg38/geneHancer/geneHancerRegElementsDoubleElite.hg38.bb
Physical chromatin interaction	Mumbach et al. ^30^	GEO: GSE101498
Physical chromatin interaction in CD4^+^	González-Serna et al. ^29^	GEO: GSM6509371
Transposase-accessible chromatin using sequencing	Calderon et al. ^31^	GEO: GSE118189http://web.stanford.edu/group/pritchardlab/dataArchive/immune_atlas_web/index.html
Naive CD4^+^ epigenomic markers	Roadmap (Kundaje et al.)^32^	GEO: GSM325895
Database of Human DNA binding sequences experiments	ReMap (Hammal et al.)^37^	https://remap.univ-amu.fr
Predicted regulatory elements	ENCODE project consortium 2020^64^	https://www.encodeproject.org
Fine-scale recombination rate	HapMap (Kong et al.)^65^	/gbdb/hg19/decode/hapMapRelease24YRIRecombMap.bw
eQTL Catalog 2023	ELIXIR Estonia (Kerimov et al.)^35^	https://elixir.ut.ee/eqtl/
Jaspar TFBS databases	Castro-Mondragon et al. ^67^	https://jaspar.elixir.no/

**Experimental models: Cell lines**		

Human: Jurkat cell line: clone E6-1	ATCC	TIB-152

**Oligonucleotides**		

Alt-R® CRISPR-Cas9 crRNA for sequences see Table S3	This paper	
Alt-R® CRISPR-Cas9 tracrRNA	Integrated DNA technologies	Cat#1072533
Alt-R® CRISPR-Cas9 Negative Control crRNA	Integrated DNA technologies	Cat#1072544
Alt-R® CRISPR-Cas9 Negative Control crRNA	Integrated DNA technologies	Cat#1072545
Alt-R® CRISPR-Cas9 Negative Control crRNA	Integrated DNA technologies	Cat#1072546
TaqMan assay SNP genotyping C_319219_10	Applied Biosystems	Cat#4351379
See Table S3 for additional oligonucleotides	N/A	N/A

**Recombinant DNA**		

pGL3-Basic vector	Promega	Cat#E1751
pRL-SV40	Promega	Cat#E2231
pGL3- Basic with 780 bp *ATP2B4* promoter	This paper	N/A
pGL3- Basic with 780 bp *ATP2B4* promoter and 610 bp ESpromoter major haplotype	This paper	N/A
pGL3- Basic with 780 bp *ATP2B4* promoter and 610 bp ESpromoter minor haplotype	This paper	N/A
pGL3- Basic with 810 bp *LAX1* promoter rs11240391-T allele T	This paper	N/A
pGL3- Basic with 810 bp *LAX1* promoter rs11240391-G allele	This paper	N/A
pGL3- Basic with 810 bp *LAX1* promoter rs11240391-T allele and 610 bp ESpromoter major haplotype	This paper	N/A
pGL3- Basic with 810 bp *LAX1* promoter rs11240391-T allele and 610 bp ESpromoter minor haplotype	This paper	N/A
pGL3- Basic with 810 bp *LAX1* promoter rs11240391-G allele and 610 bp ESpromoter major haplotype	This paper	N/A
pGL3- Basic with 810 bp *LAX1* promoter rs11240391-G allele and 610 bp ESpromoter minor haplotype	This paper	N/A
pcDNA3.1 Mammalian expression vector	ThermoFisher	Cat#V79020
c Fos (FOS) (NM_005252) Human Tagged ORF Clone	Origene	Cat#RC202597
c-Jun (JUN) (NM_002228) Human Tagged ORF Clone	Origene	Cat#RC209804

**Software and algorithms**		

ClicO FS	Cheong et al. ^62^	http://clicofs.codoncloud.com
WashU Epigenome Browser	Li et al. ^63^	http://epigenomegateway.wustl.edu/browser/
RSAT: regulatory sequence analysis tools	Santana-Garcia et al. ^66^	http://rsat.sb-roscoff.fr/
Custom Alt-R™ CRISPR-cas9 guide RNA	Integrated DNA technologies	https://eu.idtdna.com/site/order/designtool/index/CRISPR_CUSTOM
Alt-R™ CRISPR HDR design tool	Integrated DNA technologies	https://eu.idtdna.com/pages/tools/alt-r-crispr-hdr-design-tool
NEBaseChanger	New England BioLabs	https://nebasechanger.neb.com/
Primer3web version 4.1.0	Untergasser et al. ^70^	https://primer3.ut.ee/
IBM SPSS Statistics	IBM	https://www.ibm.com/support/pages/downloading-ibm-spss-statistics-27ggplot
Haploview	Barrett et al.^71^	https://www.broadinstitute.org/haploview/downloads
Flowjo	Treestar	https://www.flowjo.com/
R software V4.1.3	the R Core Team and the R Foundation for Statistical Computing	https://www.r-project.org/
R package ggplot2 v3.4.1	Wilkinson et al. ^72^	https://ggplot2.tidyverse.org/
R package ggpubr v0.6.0	R package	https://rpkgs.datanovia.com/ggpubr/

### Experimental model and subject details

#### Cell lines

Jurkat cells were cultured according to standard cell culture protocols. RPMI 1640 medium with GlutaMax (61870-044, ThermoFisher, Waltham, MA, USA) supplemented with 10% fetal bovine serum (FBS, A5256701, ThermoFisher, Waltham, MA, USA) was used as the culture medium. Cells were maintained in a 37°C incubator with 95% humidity and an atmosphere containing 5% CO2. Jurkat cells were subcultured to a density of 1 million cells per milliliter. The culture medium was refreshed every three days to ensure a constant supply of nutrients. Combined stimulation with phorbol 12-myristate 13-acetate (PMA; Sigma-Aldrich, P1585) and ionomycin (Iono; Sigma-Aldrich, I3909) was performed to activate Jurkat cells. Cells were harvested during the exponential growth phase, washed, and resuspended in a fresh medium. Subsequently, PMA, a protein kinase C activator, and ionomycin, a calcium ionophore, were added at 20 ng/mL and 2.5 μM final, respectively. The cells were incubated for a specific time in each experiment.

#### Cohort

Patients diagnosed with malaria were recruited from two Senegalese locations, Dakar Primary Hospital and Tambacounda Regional Hospital, including malaria patients (*n* = 117), with 90 cases of cerebral malaria and 27 cases of severe non-cerebral malaria, as reported previously.^60^ Control samples (*n* = 79) were recruited from healthy volunteers in Dakar. Information on age and sex are available in Table S1. Age is associated with SM as shown in Table S2. Written informed consent was obtained from each patient and accompanying family members. The research protocol was approved by the institutional research ethics committee of Cheikh Anta Diop University. On the day of admission, venous blood samples were collected alongside biological data to determine parasite density, haematology, and other relevant characteristics. The presence of *Plasmodium falciparum* was checked by examining thin and thick blood smears by at least two trained biologists before initiating antimalarial treatment. A blood smear was considered positive if asexual parasites were identified, and quantification of parasitized erythrocytes was performed by counting the number per microliter (μL) of blood.

#### PBMC isolation and cryopreservation

PBMCs were isolated from adult volunteers who donated their blood in the Occitania region in France. Donors reported feeling well and were evaluated to be in good general health by a physician at the French transfusion public service (Etablissement Français du Sang, EFS). Only blood donors who were negative for the presence of HBV, HCV, HIV, and syphilis infection were selected for this work. PBMCs were isolated from buffy coats obtained 18 to 24 h after the venipuncture and processed immediately upon receipt. Isolated PBMCs were frozen in heat-inactivated FBS (Gibco FBS, ThermoFisher) containing 10% tissue culture-grade DMSO (Sigma). Cryovials containing 20x10^6^ PBMCs were transferred at −80 °C at a cooling rate of 0.2°C–1°C/min/minute using a cryopreservation module (StrataCooler Cryo Preservation Module, Agilent Technologies) and, after 24 to 96 h, transferred to liquid nitrogen. To ensure that cellular assays could be properly interpreted and to avoid confounding factors such as poor cryopreservation of the PBMCs, we quantified cell viability after thawing and a 20 ± 4 h recovery period on an automated Cellometer auto2000 (Nexcelom) cell counter using acridine orange and propidium iodide cell staining. Subject PBMCs that did not meet the viability criteria of ≥85% were excluded from analyses. This study on PBMCs from healthy adult subjects was approved by the French South-West & Overseas Ethics Committee and registered at the French Ministry of Higher Education and Research (DC-2015-2488). Experiments were performed in agreement with the guidelines of the Declaration of Helsinki.

#### Human CD4^+^ T cell purification

Purification was performed using the EasySep Human Naive CD4^+^ T cell Isolation Kit II purchased from STEMCELL technologies using the manufacturer’s recommendations. Purity (>97%) was validated using antibodies recognizing CD3 (BD Horizon BV510 Mouse Anti-Human CD3), CD4 (CD4 Monoclonal Antibody (RPA-T4), FITC, eBioscience) and CD45RA (BD Pharmingen PE-Cy7 Mouse Anti-Human CD45RA) on the MACSQuant Analyzer 10 flow cytometer (Miltenyi Biotec) (Figure S3A).

### Method details

#### Data acquisition

The data of physical chromatin interactions were obtained from Genehancer data prediction^61^ and GEO: GSM6509371.^29^ The circos plot was built using ClicO FS.^62^ All genomic regions were obtained using WashU Epigenome Browser.^63^ ATAC-seq data from a PBMC sub-cell were obtained from Calderon et al.^31^ (GEO: GSE118189). Naive CD4^+^ epigenomic markers were obtained from Roadmap Data from GEO:GSM325895 available on WashU Epigenome Browser.^32^ ReMap data^37^ were obtained from the download page (https://remap.univ-amu.fr/download_page) using non-redundant peaks. The predicted regulatory elements come from the ENCODE project^64^ available on UCSC tracks. The recombination rate data is also available on the UCSC website and comes from the HapMap project.^65^ The eQTL data and PIP value were obtained from ELIXIR Estonia eQTL Browser.^35^

#### Motif analysis

The impact of the selected SNP on transcription factor binding sites (TFBS) was assessed with the RSAT tool,^66^ in its default settings, and using the Jaspar TFBS databases^67^ (Jaspar core non-redundant vertebrates 2020 Collection). Then, for the predicted transcription factor binding sites, the presence of transcription factor in the SNP region was confirmed using ReMap data.^37^

#### Genome editing using CRISPR-Cas9

##### CRISPR-Cas9 guide selection, single-stranded oligodeoxynucleotide donor design, and RNP complex preparation

We identified guide RNAs using computational algorithms prioritizing on-target efficacy and reduction of off-target effects using the CRISPR design tool provided by IDT (Integrated DNA Technologies), the custom Alt-R CRISPR-Cas9 guide RNA, and the Alt-R CRISPR HDR design tools. A 101-bp HDR donor oligonucleotide was also designed for CRISPR-mediated Homology Directed Repair (HDR). These chemically synthesized oligoribonucleotides were manufactured by IDT: crRNAs (35 mer with a part specific to the target DNA sequence, Alt-R CRISPR-Cas9 crRNA, IDT), universal tracrRNAs (67 mer, Alt- R CRISPR-Cas9 tracrRNA, 20 nmol, 1072533, IDT) and the 100-bp single-stranded oligodeoxynucleotide donor template (ssODN). These sequences were resuspended in IDTE buffer (pH 7.5, 11-05-01-15, IDT) to achieve a final concentration of 100 μM each. The active gRNA complex was formed by mixing 5 μL of universal tracrRNA (100 μM) with 5 μL of crRNA (100 μM) and incubated for 5 min at 95°C and then returned to room temperature. The Cas9 Ribonucleoprotein (RNP) complex was assembled *in vitro* by incubating 3.4 μL of Cas9 protein (62 μM) (Alt-R-S.p Hifi Cas9 Nuclease V3, 100 μg, 1081060, IDT) with 4.8 μL active gRNA complex (crRNA-tracrRNA) and 1.8 μL of PBS (1X).

##### Deletion of ESpromoter region in Jurkat cells

The ESpromoter deletion was performed in Jurkat cells as previously described.^17^ To generate the 506-bp genomic deletion comprising the 5 SNPs, 5 x 10^5^ Jurkat cells were electroporated by the Neon transfection system (MPK5000, Invitrogen) with 2 μL of gRNA1 RNP complex and 2 μL gRNA2 RNP complex and 2 μL of HDR enhancer. Following the transfection, the pool of transfected cells was clonally plated into 96-well plates at a limiting dilution of less than 0.5 to avoid mixed clones. Following 14 days of cell growth, the individual clone was isolated, genomic DNA was extracted and amplified by PCR and then screened for the desired deletion. DNA amplification was performed directly from the clones, previously treated with 20 μL of dilution buffer and 0.5 μL of DNA Release additive, which improved DNA release from the cells. Polymerase chain reaction (PCR) was performed using 25 μL of 2X Phire Tissue Direct PCR Master Mix containing Phire Hot Start II DNA Polymerase (Thermo Fisher Scientific, Waltham, MA, USA), 2 μL of previously prepared DNA and 1 μL of forward and reverse primer (10 μM), annealing at 60°C. Clones were screened for deletion by PCR using primers F1 and R1, followed by agarose gel electrophoresis of PCR amplicons. A short fragment of 335-bp in the presence of deletion and a long fragment of 841-bp in the absence of deletion were expected. PCR products were purified using the PCR DNA Purification kit (QIAGEN), according to the manufacturer’s instructions, and the deletion was confirmed by Sanger sequencing of PCR amplicons. One wild-type clone (WT_c_) and three 506-bp biallelic deletion clones (Δ1, Δ2, and Δ3) were selected among 457 screened clones.

##### Deletion of ESpromoter region in primary T cells

CRISPR-Cas9 assays were performed as previously described, with minor adaptations.^68^ Briefly, crRNA–tracrRNA duplexes were prepared using three different crRNAs per condition targeting the ESpromoter sequence or using negative control crRNAs (IDT, Cat# 1072544, #1072545 and #1072546). RNP complexes were then prepared by adding 90 pmol of Cas9 nuclease (IDT, Cat# 1081059) to the duplexes for 20–30 min. After adding 4μL of 100 μM. Alt-R Cas9 Electroporation Enhancer (IDT, Cat# 1075916), 1.5 million T cells per condition were transfected using the P2 Primary Cell 4D-Nucleofector X kit S (Lonza) and the EH100 program with a 4D-Nucleofector (Lonza). CD4^+^ Cells were then cultured in ImmunoCult-XF T cell Expansion Medium for 24 h supplemented with ImmunoCult Human CD3/CD28/CD2 T cell Activator (25 μL/1 million cells). To assess cytokine production, a 4-h restimulation step with PMA/ionomycin/BFA was also included.

The crRNA guides used for the CRISPR-Cas9 deletion were the same as the previous experiment in Jurkat cells, with the addition of a third guide gRNA4 to enhance deletion efficiency. Genomic DNA was extracted using the DNeasy Blood and Tissue Kits for DNA Isolation (QIAGEN), according to the manufacturer’s instructions. To confirm CRISPR-Cas9-mediated deletion, PCR was performed using classical parameters with primers flanking the deleted region at an annealing temperature of 64°C. Primers (F1 and R1) used are the same as for clone selection in Jurkat cells.

##### Single nucleotide editing in Jurkat cells

The Jurkat cell line was genotyped for rs11240391 by PCR amplification of a 527-bp fragment using primers F2 and R2, followed by Sanger sequencing. The sequence indicated that the Jurkat cell line is homozygous T/T for rs11240391. To replace, by homologous recombination, the T allele with a G allele at the rs11240391 variant located in the promoter region of the *LAX1* gene, we transfected the cells with 3.6 μL of gRNA3 RNP complex, 0.4 μL HDR enhancer (3mM) and 3.6 μL at 100 μM of ssODN in which a G allele is present in place of the T allele. The active gRNA complex and Cas9 RNP complex were made as described above and transfected by the Neon transfection system into Jurkat cells. After 48 h, the pool of transfected cells was clonally plated into 96-well plates at a limiting dilution of less than 0.5 to avoid mixed clones. Cell growth, DNA extraction, and PCR amplification using primers F2 and R2 were performed as described above. The sequence of the rs11240391 variant was determined by Sanger sequencing in different clones. After screening more than 700 clones, only two heterozygous (T/G_1_ and T/G_2_) clones were successfully obtained through HR without any additional insertions, deletions, or base modifications (Figure 5A). The limited efficiency of HR with CRISPR-Cas9 is likely attributed to the difficulty of deactivating the guide RNA (gRNA) once HR has been accomplished. Notably, the Cas9 enzyme can cleave the integrated DNA fragment again, as the protospacer adjacent motif (PAM) remains unchanged. This precaution is taken to avoid introducing a new mutation by altering the PAM sequence on the DNA donor carrying the minor allele T. We selected also a wild-type control clone (WT_HR_) exposed to the CRISPR-Cas9 complex but unedited.

#### Luciferase reporter assay

##### Promoter activity

The 780-bp *ATP2B4* promoter fragment (GRCh38, chr1: 203626081–203626860) was inserted into the MlulI-XhoI sites of the pGL3-basic vector (Promega, Madison, WI, USA, Cat#E1751), which contained the firefly luciferase coding sequence (GeneCust, Boynes, France). The 810-bp *LAX1* promoter region (GRCh38, chr1: 203764726–203765536) containing rs1124039 was also inserted into the MlulI-XhoI sites of the pGL3-basic vector (Promega, Madison, WI, USA, Cat#E1751). These constructs were supplied by Genecust custom services (Luxembourg). Site-directed mutagenesis was then performed to modify the T allele to G in rs1124039 using the Q5 Site-Directed Mutagenesis Kit (New England Biolabs) and the Forward 5’- CTGTGCCCTGgGCTTCATTAG -3′ and Reverse 5’- GAAACTCTGCTCAGCTCTTAATC -3′ mutagenesis primers designed by NEBaseChanger tool.

Jurkat cells were transfected using the Neon Transfection system (Invitrogen) according to the manufacturer’s instructions. One day before transfection, cells were diluted to a concentration of 0.6 million. In each experiment, 1 million cells were co-transfected with 1 μg of vectors: (1) negative control vector (empty pGL3-basic vector (Cat# E1751)) or the construct to be tested (pGL3-*ATP2B4* promoter, pGL3-*LAX1* rs11240391-T allele, pGL3-*LAX1* rs11240391-G allele) and (2) 200 ng of pRL-SV40 (plasmid encoding renilla luciferase from Promega (Cat#E2231)), as an internal control for transfection efficiency. After transfection, Jurkat cells were rested at 37°C in 5% CO2 for 24 h. Under stimulation conditions, Jurkat cells were treated with PMA/ionomycin and incubated for 6 or 24 h. Next, cells were subjected to firefly and renilla luciferase activity on 20 μL of cell lysate with 100μL LARII (1X) followed by 100μL of Stop and go (1X) according to the standard instructions provided in the Dual-Luciferase kit (Promega, Madison, WI, USA) using a TriStar LB 941 Multimode Microplate Reader (Berthold Technologies, Thermo Fisher Scientific, Waltham, MA, USA). The firefly luciferase activity of each sample was normalized to renilla luciferase and expressed as fold change relative to the empty vector control. Three experiments were performed in triplicate.

##### Enhancer-silencer activity

From the construct containing the 780-bp fragment of *ATP2B4* promoter (GRCh38, chr1: 203626081–203626860) at the MluI-XhoI sites, the 601-bp fragment of *ATP2B4* ESpromoter (GRCh38, chr1: 203682499–203683100) was cloned into the SalI-BamHI sites either with the minor or major alleles of the five SNPs (rs11240734, rs1541252, rs1541253, rs1541254, and rs1541255). The 601-bp fragment of *ATP2B4* ESpromoter containing the respective minor or major allele of the 5 SNPs was cloned into the SalI-BamHI sites in the construct having the 810-bp of *LAX1* promoter region (GRCh38, chr1: 203764726–203765536) at the MluI-XhoI used for the promoter activity. Luciferase assays were performed in Jurkat cells as described above.

##### Effect of transcription factors

For the luciferase assay, the cells were transfected with a total of 2 μg of plasmid, including (1) 0.5 μg of pRL-SV40 and (2) 0.5 μg of either Firefly luciferase gene pGL3-*LAX1* rs11240391-T allele or pGL3-*LAX1* rs11240391-G allele with or without (3) 0.5 μg of expression plasmids coding for proteins of interest (FOS, RC202597, Origene) and or (JUN, RC209804, Origene) completed with (4) 0–1 μg of empty pcDNA3.1 plasmid to equalize DNA quantities as previously described^69^ in each condition, as detailed below.

1: 0.5 μg pGL3-Renilla + 0.5 μg pGL3-*LAX1*-T + 1 μg pcDNA3.1
2: 0.5 μg pGL3-Renilla + 0.5 μg pGL3-*LAX1*-G + 1 μg pcDNA3.1
3: 0.5 μg pGL3-Renilla + 0.5 μg pGL3-*LAX1*-T + 0.5 μg plasmide JUN + 0.5 μg pcDNA3.1
4: 0.5 μg pGL3-Renilla + 0.5 μg pGL3-*LAX1*-G + 0.5 μg plasmide JUN + 0.5 μg pcDNA3.1
5: 0.5 μg pGL3-Renilla + 0.5 μg pGL3-*LAX1*-T + 0.5 μg plasmide FOS + 0.5 μg pcDNA3.1
6: 0.5 μg pGL3-Renilla + 0.5 μg pGL3-*LAX1*-G + 0.5 μg plasmide FOS + 0.5 μg pcDNA3.1
7: 0.5 μg pGL3-Renilla + 0.5 μg pGL3-*LAX1*-T + 0.5 μg plasmide JUN + 0.5 μg plasmide FOS
8: 0.5 μg pGL3-Renilla + 0.5 μg pGL3-*LAX1*-G + 0.5 μg plasmide JUN + 0.5 μg plasmide FOS

Luciferase assays were performed in Jurkat cells as described above.

##### Transfection of WT and T/G edited clones with transcription factors

Jurkat WT clone WT_HR_ (wild-type clone isolated after CRISPR/Cas9) and heterozygous clones T/G_1_ and T/G_2_ were counted before being diluted to 0.3 million cells per milliliter in RPMI+glutamax culture medium (10% decomplemented Fetal Bovine Serum, FBS) 24 h before transfections. Cells were washed in 10 mL of 1X DPBS. For each clone, 1 million cells resuspended in 100 μL of T buffer (NEON Invitrogen) were co-transfected with 500 ng of FOS and 500 ng of JUN expression plasmids (RC202597 and RC209804, respectively, Origene). In the condition without plasmids, the clones underwent the electric shock of transfection (1200 V, 40 ms, 1 pulse) and will serve as controls. For each sample, each condition was performed in two replicates. Cell pellets were recovered after 48h of cell culture.

##### RT-qPCR

RNA extractions from Jurkat wild-type cells and edited clones (deletion or allele modification) were conducted using the RNeasy Plus mini kit (Qiagen, Hilden, Germany). 1 μg of RNA per sample was transcribed into cDNA using the Superscript VILO Master Mix (Invitrogen, Thermo Fisher Scientific, Waltham, MA, USA). Real-time quantitative PCR (RT-qPCR) was performed using the SYBR Select Master Mix (ThermoFisher Scientific, Applied Biosystems Waltham, MA, USA) on the QuantStudio 6 Flex instrument on 10-fold diluted cDNA. Primers F3/R3 for *ATP2B4* and F4/R4 for *LAX1* gene expression quantification were designed using Primer3 software.^70^ Gene expression was normalized using the HPRT1 housekeeping gene, and the relative expression was computed using the ΔΔCT method. The data provided is an average of triplicates from three independent experiments per sample.

Approximately 50,000 T cells per condition were used, and total RNA was extracted using the NucleoSpin RNA XS kit (MACHEREY-NAGEL). Reverse transcription was carried out with the SensiFAST cDNA Synthesis Kit (Ozyme), and quantitative PCR was performed using the SsoAdvanced Universal SYBR Green Supermix on a LightCycler 480 instrument (Roche Diagnostics). Primers F5/R5 for *ATP2B4* and F4/R4 for *LAX1* gene expression quantification were used.

##### Flow cytometry staining and analyses

The day before PMA/Ionomycin stimulation, Jurkat cells and clones were diluted to a concentration of 0.6 million. The cells were then stimulated as described above. After stimulation, samples were collected at 6 time points: 0 h, every 15 min from 1 to 2 h, and a final point at 4 h. Samples were stored at 4°C until collection of the last time point at 4 h. For each time point, samples were divided into two groups: unstained cells (negative) and stained cells (positive) with an anti-CD69 antibody (310904, BioLegend). The cells were then centrifuged at 1500 rpm for 5 min at 4°C. Positive samples were resuspended with 100 μL of anti-CD69/DPBS antibody. After 30 min of incubation in the dark at 4°C, samples were washed with 3mL DPBS and centrifuged at 1500 rpm. Cells were then resuspended in 250 μL of DPBS. Negative samples were resuspended in 250 μL of DPBS. Samples were acquired on the LSRFortessa-X20 cytometer (BD Bio-sciences), and data were analyzed using FlowJo software (version 10, LLC).

After 24 h of cell culture, primary T cells were stimulated using 10 ng/mL Phorbol 12-Myristate 13-Acetate (PMA, Sigma) and 1 μg/mL Ionomycin (Sigma) for 4 h in the presence of 10 μg/mL Brefeldin A (Sigma). Cells were then labeled with the fixable viability dye eFluor 506 and stained with fluorophore-conjugated antibodies specific for CD69-BV711 (310944, BioLegend), CD25-PeCy7 (302612, BioLegend) and IL-2-BV750 (MQ1-17H12 (RUO), BD Biosciences) by using the Cytofix/Cytoperm Fixation/Permeabilization kit (BD Biosciences) according to the manufacturer’s instructions. Data acquisition was performed on FACSymphony Cell Analyzer (BD Biosciences). Analyses were carried out using FlowJo and Prism software.

##### TaqMan genotyping

Genomic DNA from the Senegalese population was extracted and amplified as described previously.^60^ The rs11240391 single nucleotide polymorphism (SNP) was genotyped using the TaqMan allelic discrimination technique (4351379, C_319219_10, Thermofisher, Waltham, MA, USA) on the QuantStudio 6 Flex instrument. A total of 117 severe malaria cases and 79 controls were genotyped. The master mix consisted of 1 μL of genomic DNA at a concentration of 12.5 ng/μL, 2.1 μL of 2x master mix (TaqMan Genotyping Master Mix, Applied Biosystems, Waltham, MA, USA), and 0.06 μL for the Taqman 40x assays in a final volume of 5 μL. The thermal cycling program included an initial step of 10 min at 95°C, followed by 40 cycles.

##### Nuclear extracts preparation and electrophoretic mobility shift assay (EMSA)

Nuclear proteins were extracted from the Jurkat cell line using the Nuclear extract kit (Active Motif, Cat#40010) according to the manufacturer’s instructions. For the preparation of nuclear extracts, 9 × 10^6^ fresh wild-type cells were used. Protein concentrations were determined by a Bradford protein assay (Bio-Rad). EMSA was performed using the Gelshift Chemiluminescent EMSA Kit (Active Motif, Cat#37341) according to manufacturer’s instructions. For the oligo probe, a 30-bp fragment with the SNP rs11240391 centered in the middle was made by annealing two biotinylated oligonucleotides (OligoB Ref F and OligoB Ref R for the major T allele; OligoB Alt F and OligoB Alt R for the minor G allele). For the competition, the same 30-bp non-biotinylated oligos were used (Oligo Ref F and Oligo Ref R for the major T allele; Oligo Alt F and Oligo Alt R for the minor G allele). The second competition was performed by using AP-1 competitor corresponding to 27-bp non-biotinylated oligos containing bases −65 to −39 of human *MMP13* promoter sequence^38^ (OligoAP-1 F and OligoAP-1 R). For each reaction, we have used 10 μg of nuclear extract, 2 μL of 0.2 μM of biotinylated oligo duplexes, and 2 μL of 40 μM of non-biotinylated competitor oligo duplexes. Complexes were separated on 6% polyacrylamide Novex DNA Retardation Gels prepared with 0.5X TBE gel buffer.

### Quantification and statistical analysis

#### Statistical analyses

The Haploview tool^71^ was used to assess the deviation of genotype frequency from Hardy Weinberg equilibrium in the control group. The observed genotype frequencies were consistent with Hardy–Weinberg equilibrium. Genetic association analyses were conducted using IBM SPSS Statistics 27 (IBM, NY, USA). Logistic regression analyses were performed to adjust for the influence of age and to determine genetic interactions between *ATP2B4* and *LAX1* gene variants. Haplotype frequency estimation and haplotype association tests were performed with Haploview^71^ using the “do association test” option and the “Case-control data” parameter. Statistical analyses of luciferase reporter assays and RT-qPCR results were performed by Student’s t-tests (ns: not significant, ∗*p* < 0.05, ∗∗*p* < 0.01, ∗∗∗*p* < 0.001, ∗∗∗∗*p* < 0.0001). All tests were two-sided. Graphs were generated using ggplot2^72^ and ggpubr. Paired t-test was used to compare the percentage of CD69-positive cells of wild-type cells with that of cells mutated with the CRISPR-Cas9 method, after assessing the normality of the data using the Shapiro-Wilk method. These tests were one-sided, with an alternative hypothesis based on known *LAX1* expression in the cells and the known effect of *LAX1* on CD69 expression.

## Supplemental information

Document S1. Figures S1–S5 and Tables S2 and S3

Table S1. Description of the case-control cohort recruited in Senegal, related to Figure 6 For each individual, the details indicated are identification number, phenotype (control [Ctrl]; Severe malaria: SM), age in years, sex (female [F], male [M]), and the genotype for rs11240391

Document S2. Article plus supplemental information

## References

[1] Demircioğlu D., Cukuroglu E., Kindermans M., Nandi T., Calabrese C., Fonseca N.A., Kahles A., Lehmann K.-V., Stegle O., Brazma A. (2019). A Pan-cancer Transcriptome Analysis Reveals Pervasive Regulation through Alternative Promoters. Cell.

[2] Zhuang H.-H., Qu Q., Teng X.-Q., Dai Y.-H., Qu J. (2023). Superenhancers as master gene regulators and novel therapeutic targets in brain tumors. Exp. Mol. Med..

[3] Sun N., Akay L.A., Murdock M.H., Park Y., Galiana-Melendez F., Bubnys A., Galani K., Mathys H., Jiang X., Ng A.P. (2023). Single-nucleus multiregion transcriptomic analysis of brain vasculature in Alzheimer’s disease. Nat. Neurosci..

[4] Riboldi G.M., Vialle R.A., Navarro E., Udine E., de Paiva Lopes K., Humphrey J., Allan A., Parks M., Henderson B., Astudillo K. (2022). Transcriptome deregulation of peripheral monocytes and whole blood in GBA-related Parkinson’s disease. Mol. Neurodegener..

[5] Wilk A.J., Lee M.J., Wei B., Parks B., Pi R., Martínez-Colón G.J., Ranganath T., Zhao N.Q., Taylor S., Becker W. (2021). Multi-omic profiling reveals widespread dysregulation of innate immunity and hematopoiesis in COVID-19. J. Exp. Med..

[6] Cabantous S., Doumbo O., Poudiougou B., Louis L., Barry A., Oumar A.A., Traore A., Marquet S., Dessein A. (2017). Gene Expression Analysis Reveals Genes Common to Cerebral Malaria and Neurodegenerative Disorders. J. Infect. Dis..

[7] Cabantous S., Poudiougou B., Bergon A., Barry A., Oumar A.A., Traore A.M., Chevillard C., Doumbo O., Dessein A., Marquet S. (2020). Understanding Human Cerebral Malaria through a Blood Transcriptomic Signature: Evidences for Erythrocyte Alteration, Immune/Inflammatory Dysregulation, and Brain Dysfunction. Mediat. Inflamm..

[8] Nakano M., Ota M., Takeshima Y., Iwasaki Y., Hatano H., Nagafuchi Y., Itamiya T., Maeda J., Yoshida R., Yamada S. (2022). Distinct transcriptome architectures underlying lupus establishment and exacerbation. Cell.

[9] Reyes M., Filbin M.R., Bhattacharyya R.P., Billman K., Eisenhaure T., Hung D.T., Levy B.D., Baron R.M., Blainey P.C., Goldberg M.B., Hacohen N. (2020). An immune-cell signature of bacterial sepsis. Nat. Med..

[10] Zubovic L., Piazza S., Tebaldi T., Cozzuto L., Palazzo G., Sidarovich V., De Sanctis V., Bertorelli R., Lammens T., Hofmans M. (2020). The altered transcriptome of pediatric myelodysplastic syndrome revealed by RNA sequencing. J. Hematol. Oncol..

[11] Gentles A.J., Newman A.M., Liu C.L., Bratman S.V., Feng W., Kim D., Nair V.S., Xu Y., Khuong A., Hoang C.D. (2015). The prognostic landscape of genes and infiltrating immune cells across human cancers. Nat. Med..

[12] Wang Q., Zhang Y., Wang M., Song W.-M., Shen Q., McKenzie A., Choi I., Zhou X., Pan P.-Y., Yue Z., Zhang B. (2019). The landscape of multiscale transcriptomic networks and key regulators in Parkinson’s disease. Nat. Commun..

[13] Hao Y., Hao S., Andersen-Nissen E., Mauck W.M., Zheng S., Butler A., Lee M.J., Wilk A.J., Darby C., Zager M. (2021). Integrated analysis of multimodal single-cell data. Cell.

[14] Pennacchio L.A., Bickmore W., Dean A., Nobrega M.A., Bejerano G. (2013). Enhancers: five essential questions. Nat. Rev. Genet..

[15] Dao L.T.M., Spicuglia S. (2018). Transcriptional regulation by promoters with enhancer function. Transcription.

[16] Santiago-Algarra D., Souaid C., Singh H., Dao L.T.M., Hussain S., Medina-Rivera A., Ramirez-Navarro L., Castro-Mondragon J.A., Sadouni N., Charbonnier G., Spicuglia S. (2021). Epromoters function as a hub to recruit key transcription factors required for the inflammatory response. Nat. Commun..

[17] Nisar S., Torres M., Thiam A., Pouvelle B., Rosier F., Gallardo F., Ka O., Mbengue B., Diallo R.N., Brosseau L. (2022). Identification of ATP2B4 Regulatory Element Containing Functional Genetic Variants Associated with Severe Malaria. Int. J. Mol. Sci..

[18] Malfait J., Wan J., Spicuglia S. (2023). Epromoters are new players in the regulatory landscape with potential pleiotropic roles. Bioessays.

[19] Zabidi M.A., Arnold C.D., Schernhuber K., Pagani M., Rath M., Frank O., Stark A. (2015). Enhancer-core-promoter specificity separates developmental and housekeeping gene regulation. Nature.

[20] Malfait J., Wan J., Singh H., Souaid C., Farah G., Esnault C., Sarrazin S., Sieweke M., Spicuglia S. (2024). Epromoters bind key stress-related transcription factors to regulate clusters of stress response genes. bioRxiv.

[21] Buniello A., MacArthur J.A.L., Cerezo M., Harris L.W., Hayhurst J., Malangone C., McMahon A., Morales J., Mountjoy E., Sollis E. (2019). The NHGRI-EBI GWAS Catalog of published genome-wide association studies, targeted arrays and summary statistics 2019. Nucleic Acids Res..

[22] Boix C.A., James B.T., Park Y.P., Meuleman W., Kellis M. (2021). Regulatory genomic circuitry of human disease loci by integrative epigenomics. Nature.

[23] Adjemout M., Pouvelle B., Thiam F., Thiam A., Torres M., Nisar S., Mbengue B., Dieye A., Rihet P., Marquet S. (2023). Concurrent PIEZO1 activation and ATP2B4 blockade effectively reduce the risk of cerebral malaria and inhibit in vitro Plasmodium falciparum multiplication in red blood cells. Genes Dis..

[24] Timmann C., Thye T., Vens M., Evans J., May J., Ehmen C., Sievertsen J., Muntau B., Ruge G., Loag W. (2012). Genome-wide association study indicates two novel resistance loci for severe malaria. Nature.

[25] Band G., Le Q.S., Jostins L., Pirinen M., Kivinen K., Jallow M., Sisay-Joof F., Bojang K., Pinder M., Sirugo G. (2013). Imputation-based meta-analysis of severe malaria in three African populations. PLoS Genet..

[26] Band G., Rockett K.A., Spencer C.C.A., Kwiatkowski D.P., Malaria Genomic Epidemiology Network (2015). A novel locus of resistance to severe malaria in a region of ancient balancing selection. Nature.

[27] Ravenhall M., Campino S., Sepúlveda N., Manjurano A., Nadjm B., Mtove G., Wangai H., Maxwell C., Olomi R., Reyburn H. (2018). Novel genetic polymorphisms associated with severe malaria and under selective pressure in North-eastern Tanzania. PLoS Genet..

[28] Malaria Genomic Epidemiology Network (2019). Insights into malaria susceptibility using genome-wide data on 17,000 individuals from Africa, Asia and Oceania. Nat. Commun..

[29] González-Serna D., Shi C., Kerick M., Hankinson J., Ding J., McGovern A., Tutino M., Villanueva-Martin G., Ortego-Centeno N., Callejas J.L. (2023). Identification of Mechanisms by Which Genetic Susceptibility Loci Influence Systemic Sclerosis Risk Using Functional Genomics in Primary T Cells and Monocytes. Arthritis Rheumatol..

[30] Mumbach M.R., Satpathy A.T., Boyle E.A., Dai C., Gowen B.G., Cho S.W., Nguyen M.L., Rubin A.J., Granja J.M., Kazane K.R. (2017). Enhancer connectome in primary human cells identifies target genes of disease-associated DNA elements. Nat. Genet..

[31] Calderon D., Nguyen M.L.T., Mezger A., Kathiria A., Müller F., Nguyen V., Lescano N., Wu B., Trombetta J., Ribado J.V. (2019). Landscape of stimulation-responsive chromatin across diverse human immune cells. Nat. Genet..

[32] Kundaje A., Meuleman W., Ernst J., Bilenky M., Yen A., Heravi-Moussavi A., Kheradpour P., Zhang Z., Wang J., Roadmap Epigenomics Consortium (2015). Integrative analysis of 111 reference human epigenomes. Nature.

[33] Merino-Wong M., Niemeyer B.A., Alansary D. (2021). Plasma Membrane Calcium ATPase Regulates Stoichiometry of CD4+ T-Cell Compartments. Front. Immunol..

[34] Zhu M., Granillo O., Wen R., Yang K., Dai X., Wang D., Zhang W. (2005). Negative regulation of lymphocyte activation by the adaptor protein LAX. J. Immunol..

[35] Kerimov N., Tambets R., Hayhurst J.D., Rahu I., Kolberg P., Raudvere U., Kuzmin I., Chowdhary A., Vija A., Teras H.J. (2023). eQTL Catalogue 2023: New datasets, X chromosome QTLs, and improved detection and visualisation of transcript-level QTLs. PLoS Genet..

[36] Kwong A., Boughton A.P., Wang M., VandeHaar P., Boehnke M., Abecasis G., Kang H.M. (2022). FIVEx: an interactive eQTL browser across public datasets. Bioinformatics.

[37] Hammal F., de Langen P., Bergon A., Lopez F., Ballester B. (2022). ReMap 2022: a database of Human, Mouse, Drosophila and Arabidopsis regulatory regions from an integrative analysis of DNA-binding sequencing experiments. Nucleic Acids Res..

[38] Samuel S., Beifuss K.K., Bernstein L.R. (2007). YB-1 binds to the MMP-13 promoter sequence and represses MMP-13 transactivation via the AP-1 site. Biochim. Biophys. Acta.

[39] Moss P. (2022). The T cell immune response against SARS-CoV-2. Nat. Immunol..

[40] Nlinwe O.N., Kusi K.A., Adu B., Sedegah M. (2018). T-cell responses against Malaria: Effect of parasite antigen diversity and relevance for vaccine development. Vaccine.

[41] Kurup S.P., Butler N.S., Harty J.T. (2019). T cell-mediated immunity to malaria. Nat. Rev. Immunol..

[42] Machiela M.J., Chanock S.J. (2015). LDlink: a web-based application for exploring population-specific haplotype structure and linking correlated alleles of possible functional variants. Bioinformatics.

[43] Ogbourne S., Antalis T.M. (1998). Transcriptional control and the role of silencers in transcriptional regulation in eukaryotes. Biochem. J..

[44] Jiang J., Cai H., Zhou Q., Levine M. (1993). Conversion of a dorsal-dependent silencer into an enhancer: evidence for dorsal corepressors. EMBO J..

[45] Gisselbrecht S.S., Palagi A., Kurland J.V., Rogers J.M., Ozadam H., Zhan Y., Dekker J., Bulyk M.L. (2020). Transcriptional Silencers in Drosophila Serve a Dual Role as Transcriptional Enhancers in Alternate Cellular Contexts. Mol. Cell.

[46] Halfon M.S. (2020). Silencers, Enhancers, and the Multifunctional Regulatory Genome. Trends Genet..

[47] Kehayova P., Monahan K., Chen W., Maniatis T. (2011). Regulatory elements required for the activation and repression of the protocadherin-alpha gene cluster. Proc. Natl. Acad. Sci. USA.

[48] Huang D., Ovcharenko I. (2022). Enhancer-silencer transitions in the human genome. Genome Res..

[49] Wei X., Xiang Y., Peters D.T., Marius C., Sun T., Shan R., Ou J., Lin X., Yue F., Li W. (2022). HiCAR is a robust and sensitive method to analyze open-chromatin-associated genome organization. Mol. Cell.

[50] Hussain S., Sadouni N., van Essen D., Dao L.T.M., Ferré Q., Charbonnier G., Torres M., Gallardo F., Lecellier C.-H., Sexton T. (2023). Short tandem repeats are important contributors to silencer elements in T cells. Nucleic Acids Res..

[51] Spitz F., Furlong E.E.M. (2012). Transcription factors: from enhancer binding to developmental control. Nat. Rev. Genet..

[52] Yagi R., Zhu J., Paul W.E. (2011). An updated view on transcription factor GATA3-mediated regulation of Th1 and Th2 cell differentiation. Int. Immunol..

[53] Doni Jayavelu N., Jajodia A., Mishra A., Hawkins R.D. (2020). Candidate silencer elements for the human and mouse genomes. Nat. Commun..

[54] Shi Y., Seto E., Chang L.S., Shenk T. (1991). Transcriptional repression by YY1, a human GLI-Krüppel-related protein, and relief of repression by adenovirus E1A protein. Cell.

[55] Caride A.J., Filoteo A.G., Penheiter A.R., Pászty K., Enyedi A., Penniston J.T. (2001). Delayed activation of the plasma membrane calcium pump by a sudden increase in Ca2+: fast pumps reside in fast cells. Cell Calcium.

[56] Supper V., Schiller H.B., Paster W., Forster F., Boulègue C., Mitulovic G., Leksa V., Ohradanova-Repic A., Machacek C., Schatzlmaier P. (2016). Association of CD147 and Calcium Exporter PMCA4 Uncouples IL-2 Expression from Early TCR Signaling. J. Immunol..

[57] Zhu M., Janssen E., Leung K., Zhang W. (2002). Molecular cloning of a novel gene encoding a membrane-associated adaptor protein (LAX) in lymphocyte signaling. J. Biol. Chem..

[58] Zaugg J.B., Sahlén P., Andersson R., Alberich-Jorda M., de Laat W., Deplancke B., Ferrer J., Mandrup S., Natoli G., Plewczynski D. (2022). Current challenges in understanding the role of enhancers in disease. Nat. Struct. Mol. Biol..

[59] Gupta R.M., Hadaya J., Trehan A., Zekavat S.M., Roselli C., Klarin D., Emdin C.A., Hilvering C.R.E., Bianchi V., Mueller C. (2017). A Genetic Variant Associated with Five Vascular Diseases Is a Distal Regulator of Endothelin-1 Gene Expression. Cell.

[60] Thiam A., Baaklini S., Mbengue B., Nisar S., Diarra M., Marquet S., Fall M.M., Sanka M., Thiam F., Diallo R.N. (2018). NCR3 polymorphism, haematological parameters, and severe malaria in Senegalese patients. PeerJ.

[61] Fishilevich S., Nudel R., Rappaport N., Hadar R., Plaschkes I., Iny Stein T., Rosen N., Kohn A., Twik M., Safran M. (2017). GeneHancer: genome-wide integration of enhancers and target genes in GeneCards. Database.

[62] Cheong W.-H., Tan Y.-C., Yap S.-J., Ng K.-P. (2015). ClicO FS: an interactive web-based service of Circos. Bioinformatics.

[63] Li D., Purushotham D., Harrison J.K., Hsu S., Zhuo X., Fan C., Liu S., Xu V., Chen S., Xu J. (2022). WashU Epigenome Browser update 2022. Nucleic Acids Res..

[64] Moore J.E., Purcaro M.J., Pratt H.E., Epstein C.B., Shoresh N., Adrian J., Kawli T., Davis C.A., Dobin A., ENCODE Project Consortium (2020). Expanded encyclopaedias of DNA elements in the human and mouse genomes. Nature.

[65] Kong A., Thorleifsson G., Gudbjartsson D.F., Masson G., Sigurdsson A., Jonasdottir A., Walters G.B., Jonasdottir A., Gylfason A., Kristinsson K.T. (2010). Fine-scale recombination rate differences between sexes, populations and individuals. Nature.

[66] Santana-Garcia W., Castro-Mondragon J.A., Padilla-Gálvez M., Nguyen N.T.T., Elizondo-Salas A., Ksouri N., Gerbes F., Thieffry D., Vincens P., Contreras-Moreira B. (2022). RSAT 2022: regulatory sequence analysis tools. Nucleic Acids Res..

[67] Castro-Mondragon J.A., Riudavets-Puig R., Rauluseviciute I., Lemma R.B., Turchi L., Blanc-Mathieu R., Lucas J., Boddie P., Khan A., Manosalva Pérez N. (2022). JASPAR 2022: the 9th release of the open-access database of transcription factor binding profiles. Nucleic Acids Res..

[68] Seki A., Rutz S. (2018). Optimized RNP transfection for highly efficient CRISPR/Cas9-mediated gene knockout in primary T cells. J. Exp. Med..

[69] Canac R., Cimarosti B., Girardeau A., Forest V., Olchesqui P., Poschmann J., Redon R., Lemarchand P., Gaborit N., Lamirault G. (2022). Deciphering Transcriptional Networks during Human Cardiac Development. Cells.

[70] Untergasser A., Cutcutache I., Koressaar T., Ye J., Faircloth B.C., Remm M., Rozen S.G. (2012). Primer3--new capabilities and interfaces. Nucleic Acids Res..

[71] Barrett J.C., Fry B., Maller J., Daly M.J. (2005). Haploview: analysis and visualization of LD and haplotype maps. Bioinformatics.

[72] Wilkinson L. (2011). ggplot2: Elegant Graphics for Data Analysis by WICKHAM, H. Biometrics.

