# Supplementary material for: Unraveling an enhancer-silencer regulatory element showing epistatic interaction with a variant that escaped genome-wide association studies
Source: Cell Genom. 2025 May 28;5(7):100889. doi: 10.1016/j.xgen.2025.100889 (PMC12278644; doi:10.1016/j.xgen.2025.100889)
Supplement: Document S1. Figures S1–S5 and Tables S2 and S3 [file mmc1.pdf]

Cell Genomics, Volume *5*

Supplemental information

Unraveling an enhancer-silencer regulatory element showing epistatic interaction with a variant that escaped genome-wide association studies

Mathieu Adjemout, Samia Nisar, Amélie Escandell, Romain Torres, Magali Torres, Hong Thu Nguyen Huu, Alassane Thiam, Iris Manosalva, Babacar Mbengue, Alioune Dieye, Véronique Adoue, Salvatore Spicuglia, Pascal Rihet, and Sandrine Marquet

Supplemental Information PDF file

Figure S1-S5

Table S2: Results of epistatic interaction between the 5 SNPs at ESpromoter and rs11240391, related to Figure 6

Table S3: Details of oligonucleotides used (name, source, identifier, and sequence) related to the Key resources table

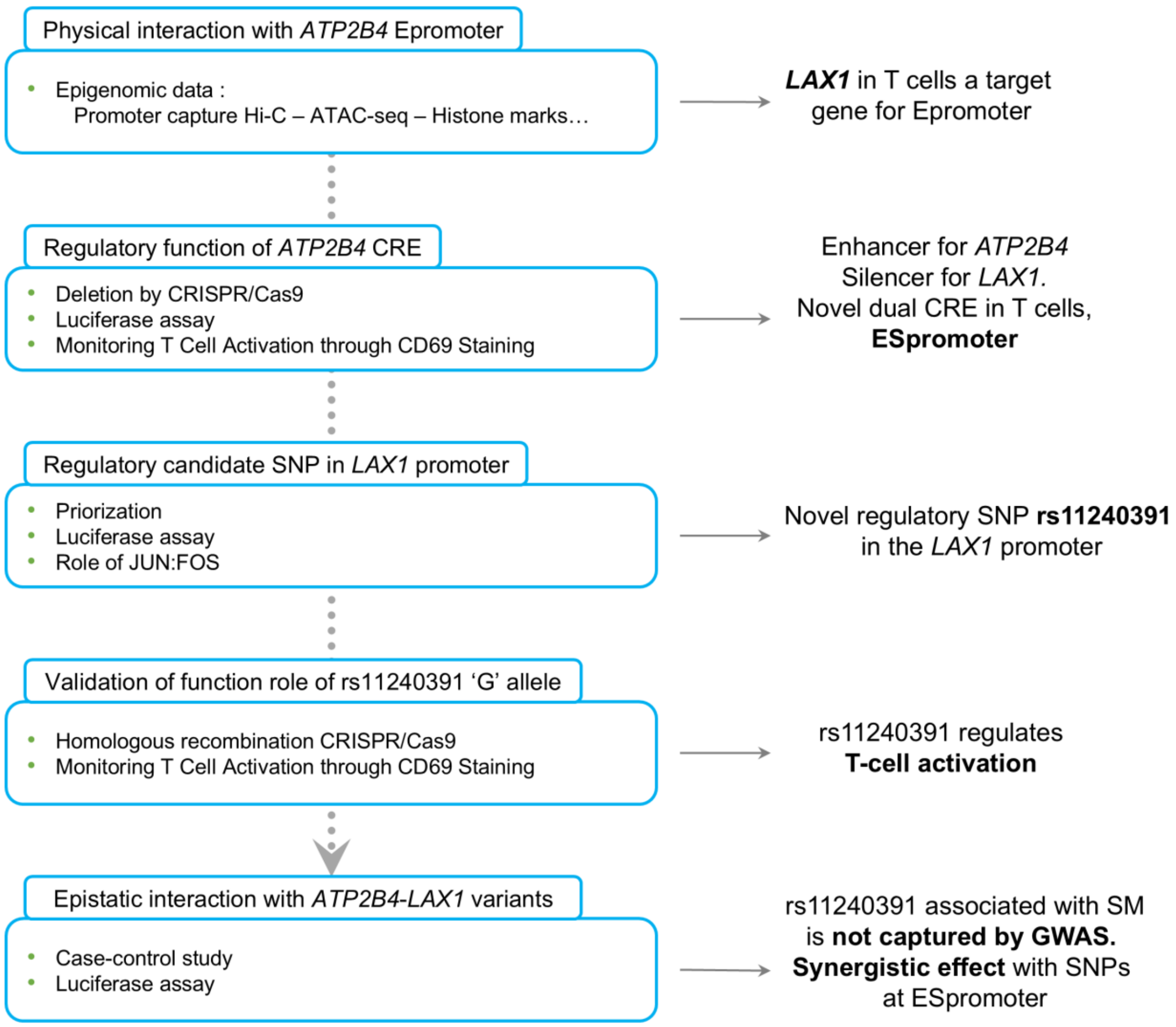

Figure S1: Workflow of bioinformatics and experimental analysis with the key findings, Related to Figure 1-6

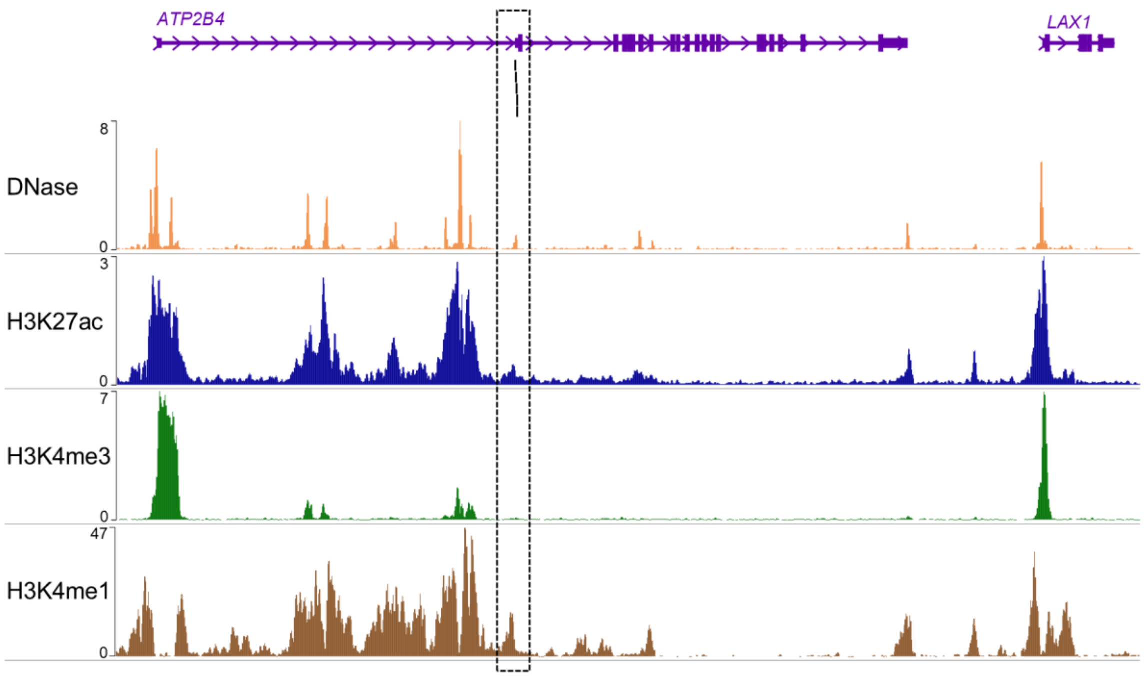

Figure S2: Epigenomic marks of Jurkat cell, Related to Figure 1

WashU epigenome browser view of epigenomic data in Jurkat cells. The frame corresponds to the Epromoter. Black lines correspond to the 5 SNPs previously identified (rs11240734, rs1541252, rs1541253, rs1541254, and rs1541255).

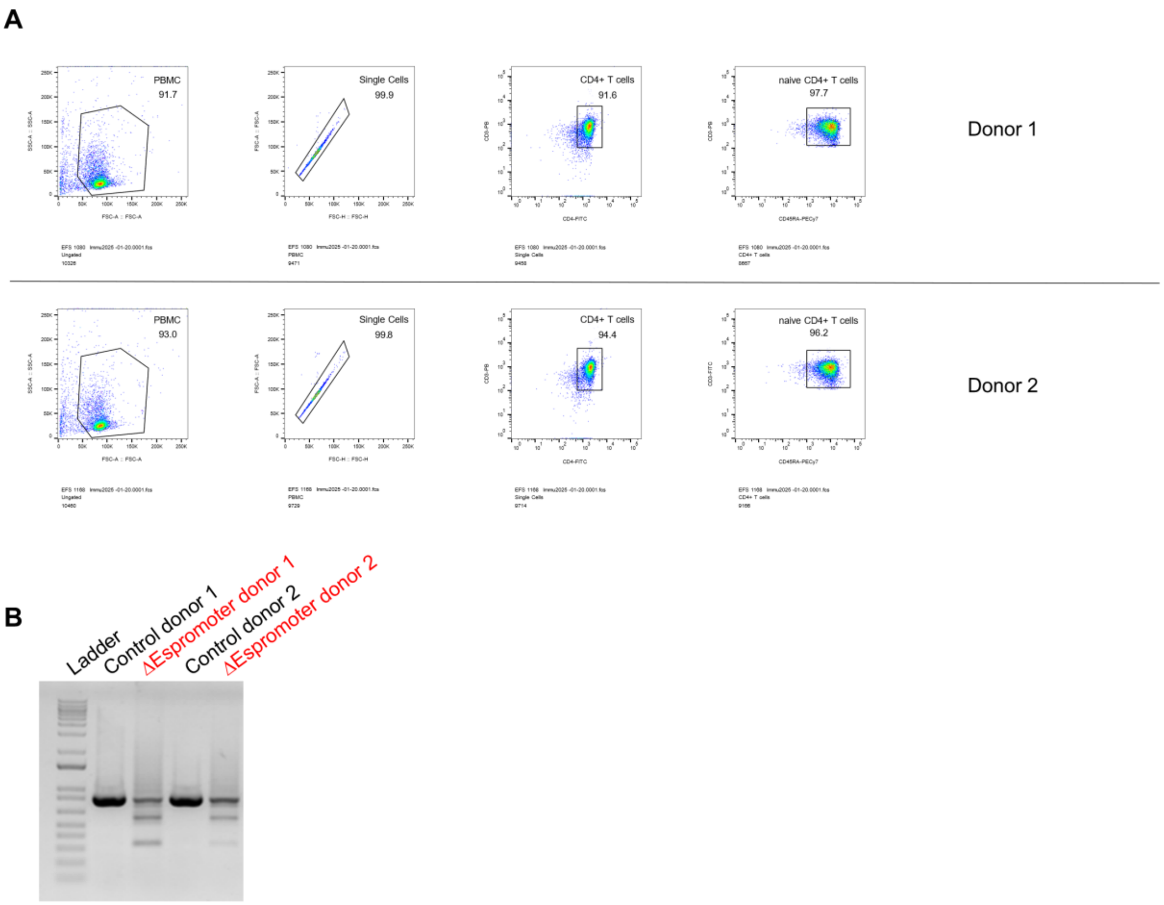

Figure S3: ESpromoter is essential for normal human primary T cell activation, Related to Figure 3.

(A) Flow cytometry gating strategy used to validate the purity of naive CD4+ T cells from each blood donor.

(B) Agarose gel electrophoresis of PCR amplification from genomic DNA extracted from cells subjected to CRISPR-Cas9-mediated ESpromoter deletion or control conditions. A smear indicates successful deletion, reflecting micro-insertions and deletions (indels) generated between the three crRNA guide sites.

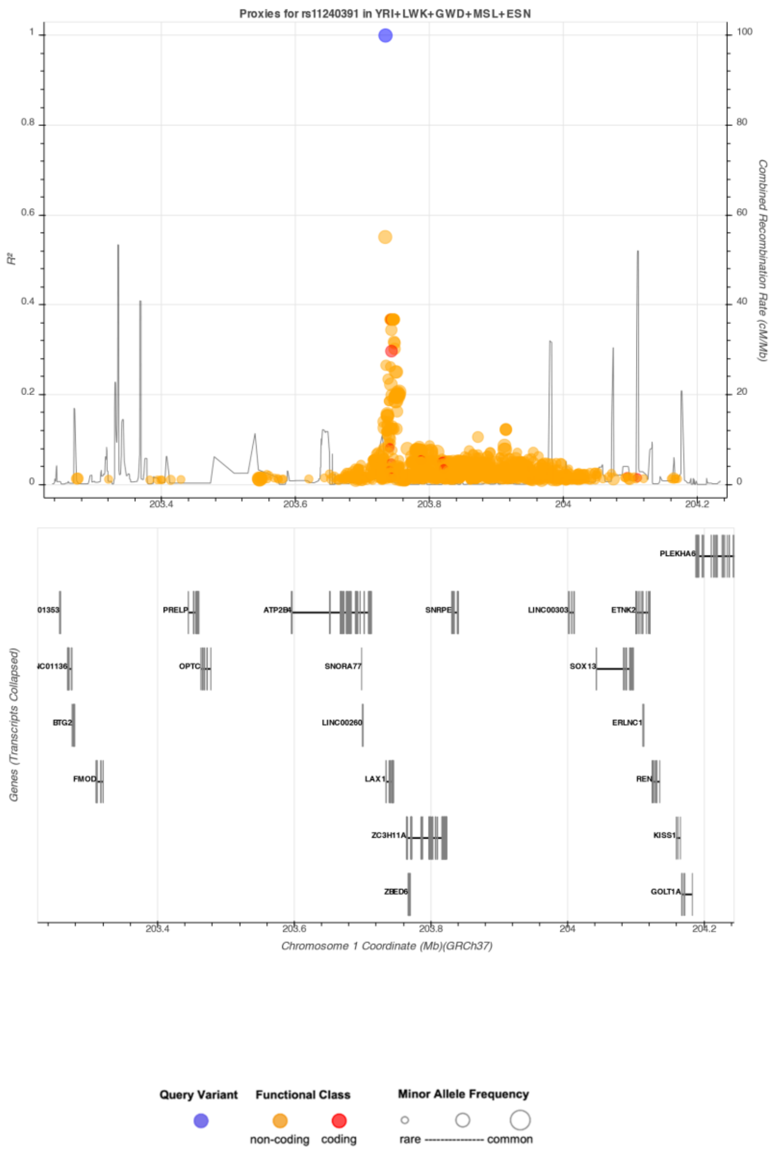

Figure S4: rs11240391 shows no significant linkage disequilibrium with nearby variants, Related to Figure 6.

The top panel is a LocusZoom plot illustrating the linkage disequilibrium (LD) along chromosome 1 (chr1:203234231-204234231-GRCh37) in African populations (YRI, LWK, GWD, MSL, ESN). The X-axis represents genomic coordinates, while the left Y-axis shows the LD (r^2) between rs11240391 and surrounding variants. The right Y-axis represents combined recombination rates estimated from HapMap data. Each point represents a genetic variant, with blue indicating the rs11240391 query variant, orange for non-coding variants, and red for coding variants. The size of each circle corresponds to the minor allele frequency (MAF), with larger circles representing more common variants. The bottom panel displays the annotated genes in the region.

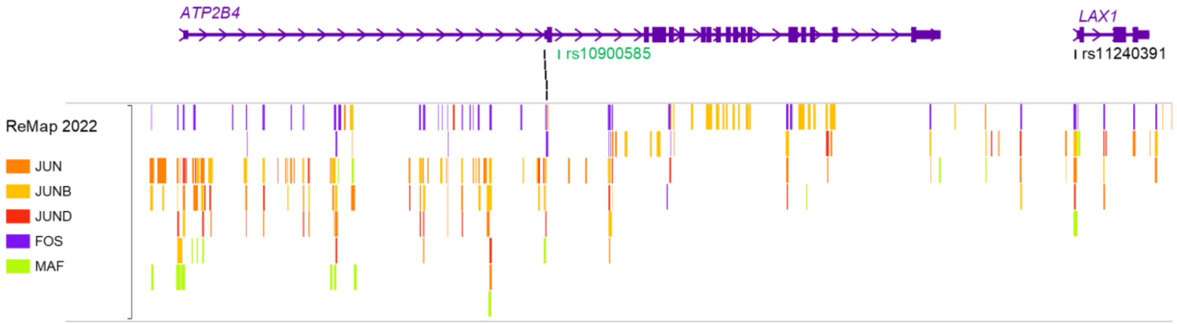

Figure S5: Identification of the FOS and JUN binding site on ESpromoter region, *ATP2B4* and *LAX1* promoters, Related to Figure 4

CHIP-seq peaks from ReMap2022 confirmed the binding of transcription factors, identified by RSAT, within the ESpromoter and *ATP2B4* and *LAX1* promoters, particularly the FOS::JUN dimer corresponding to AP-1.

	Ctrl	SM	Odd ratio (95% CI)	P-value (two-sided)
**Age**	NA	NA	0.98 (0.97-0.99)	0.002
**Sex**				
Male	40	72	1	-
Female	38	38	0.57 (0.31-1.01)	0.07
LAX1				
Risk genotypes (GG)			1	-
Protective genotype (TT+ TG)	62	71	0.38 (0.19-0.75)	0.005
ATP2B4				
Risk haplotype (Major/Major)	26	64	1	-
Protective haplotypes (Major/Minor + Minor/Minor)	49	49	0.40 (0.22-0.74)	0.004
**Combination**				
Risk genotypes *LAX1*/haplotype *ATP2B4*	6	24	1	-
Protective genotypes *LAX1*/haplotype *ATP2B4*	39	28	0.18 (0.07-0.50)	0.0008
Interaction *LAX1* × *ATP2B4*			0.33 (0.19-0.57)	0.00006

Table S2: Results of epistatic interaction between the 5 SNPs at ESpromoter and rs11240391, Related to Figure 6

Oligonucleotides		
sgRNA	SEQUENCE	PAM
gRNA1	TCCTCTACATTGGAGTTTAC	AGG
gRNA2	TAGACTTCGGACGGCTACTC	GGG
gRNA3	CCAATGTGCTAATGAAGCAC	AGG
gRNA4	CAACTGTAGTAGTAGACGTC	AGG
ssODN sequence	SEQUENCE	
ssODN – G at rs11240391	TATGTTTTCTTCTAGCAGATTAAGAGCTGAGCAGAGTTTCCTGTGCCCTGGGCTTCATTAGCACATTGGTGGTGTCGTTTCCGGTGACTGACTCTCTGTTT	
Primers for EMSA	SEQUENCE	
OligoB Ref F	5'[BIO]CCTGTGCCCTG**T**GCTTCATTAGCACATTGG 3'	
OligoB Ref R	5'[BIO]CCAATGTGCTAATGAAGC**A**CAGGGCACAGG 3'	
OligoB Alt F	5'[BIO]CCTGTGCCCTG**G**GCTTCATTAGCACATTGG 3'	
OligoB Alt R	5'[BIO]CCAATGTGCTAATGAAGC**C**CAGGGCACAGG 3'	
Oligo Ref F	5'CCTGTGCCCTG**T**GCTTCATTAGCACATTGG 3'	
Oligo Ref R	5'CCAATGTGCTAATGAAGC**A**CAGGGCACAGG 3'	
Oligo Alt F	5'CCTGTGCCCTG**G**GCTTCATTAGCACATTGG 3'	
Oligo Alt R	5'CCAATGTGCTAATGAAGC**C**CAGGGCACAGG 3'	
OligoAP-1 F	5′ CCTATCCATAAGTGA**TGACTCA**CCATT 3′	
OligoAP-1 R	5'AATGG**TGAGTCA**TCACTTATGGATAGG 3'	
PCR primers	SEQUENCE	
F1	GGCCACCCTTCAGATCACTT	
R1	GCCTCCCTGTCTCAACTTCT	
F2	TGAATCAGAAGAGGGTCCCG	
R2	CGATCTCACCGGACATGGT	
F3	CAAGAGTCTGGCCCGAGTTA	
R3	TGGACGATCTCAACAGCAGA	
F4	AGAAATTCTGAGAGCCCGGAG	
R4	GATACCCACCGCGTACTCTG	
F5	TTGGAGAACTTCTGTGGGGC	
R5	GGCATCCTTGGTGATCTCCT	

HPRT1 forward	GGGTGTTTATTCCTCATGGAC	
HPRT1 reverse	CTCCCATCTCCTTCATCACA	
Primers for mutagenesis	SEQUENCE	
Forward at rs11240391	CTGTGCCCTGgGCTTCATTAG	
Reverse at rs11240391	GAAACTCTGCTCAGCTCTTAATC	

Table S3: Details of oligonucleotides used (name, source, identifier, and sequence), Related to the Key resources table
